# Resonance-Driven Discrete Growth and Chemical Reactivity of Optically Levitated Droplets

**DOI:** 10.34133/research.0813

**Published:** 2023-08-05

**Authors:** Kaiqi Zhang, Grégory David, Yue Zhao, Hua-Yun Xiao, Ruth Signorell, Chenxi Li

**Affiliations:** ^1^ School of Environmental Science and Engineering, Shanghai Jiao Tong University, 200240 Shanghai, China.; ^2^ Laboratory of Physical Chemistry, ETH Zürich, CH-8093 Zürich, Switzerland.; ^3^ School of Agriculture and Biology, Shanghai Jiao Tong University, 200240 Shanghai, China.

## Abstract

Optical levitation provides a powerful platform for probing the physicochemical properties of nano- and microparticles. In optical levitation experiments involving nonreacting droplets, metastable states, or so-called “thermally locked” states, can emerge. However, there has been no report on thermal locking induced by chemical reactions or the impact of thermal locking on the reaction mechanisms or rates. Herein, we investigate the growth of optically levitated aqueous droplets in which sulfate forms through the SO_2_-NO_2_ and the SO_2_-Mn^2+^-O_2_ heterogeneous reactions—2 environmentally important sulfate formation pathways. We observe (semi-)discrete droplet growth occurring via consecutive thermally locked states, which result from the competition between water vapor condensation driven by sulfate formation and evaporation driven by droplet heating through resonant absorption of the trapping laser. By combining Mie theory-based analysis of the stimulated Raman scattering and droplet thermodynamics, we develop an approach to retrieve the key properties (e.g., temperature, pH, and molality) of thermally locked droplets and demonstrate that chemistry-driven thermal locking results in a signature particle growth pattern. Comparison of sulfate formation rates in locked versus unlocked droplets further reveals that thermal locking can accelerate chemical reactions or even change the dominant mechanism by promoting photoinduced reaction pathways. As light intensity enhancement within the droplet is localized near the droplet surface, the photoinduced reactions lead to droplet growth patterns similar to those driven by surface reactions. This work uncovers a novel phenomenon emerging from light–droplet interactions, offering a mechanistic framework for leveraging thermal locking to probe droplet properties and study chemical reactions under resonant conditions.

## Introduction

The interaction between light and droplets or aerosol particles plays a crucial role in Earth’s radiation balance and underpins various particle characterization techniques across scientific disciplines. Due to their small size, nano- and microparticles can exhibit phenomena distinct from bulk materials when irradiated, including quantum confinement [1], localized surface plasmon resonance [2], whispering gallery modes (WGMs) [3], optical trapping or binding [4], and light-driven propulsion [5]. Utilizing the balance of optical forces, aerosol optical tweezers (AOTs) have emerged as a powerful technique to characterize aerosol particles and droplets. By creating an optical potential well through focused laser beams, AOTs can effectively levitate single or multiple nano- to micro-sized droplets in a gas for extended periods of time [4,6]. This unique feature empowers researchers to explore droplet physicochemical properties at the single-droplet level under precisely controlled conditions [7–13].

When resonant with the trapping laser, AOT-suspended droplets can exhibit a distinctive form of metastability known as “thermal locking” [14]. The term “lock” signifies that the droplet size remains nearly constant despite external triggers for size change, e.g., variations of ambient relative humidity (RH) or trapping laser power. This phenomenon has been documented in investigations involving aqueous salt-containing droplets [9,12,14–16], where the droplet size exhibited intermittent stages of continuous shifts and near stationarity. Thermal locking occurs when the trapping laser wavelength is commensurate with a WGM of the droplet. The droplet acts as a high-quality resonant cavity that markedly extends the optical pathlength of the incident light within the droplet, leading to palpable droplet heating despite low droplet absorbance at the laser wavelength. Droplet heating elevates the water evaporation rates and counteracts triggers for droplet growth, e.g., a rising RH in the environment. Consequently, the net transfer rate of water to or from the droplet is slow and the droplet stays at a near-constant size [15–17].

To date, the thermal locking phenomenon has been induced solely by physical factors, such as variations in RH or laser power, for nonreacting droplets. By modulating droplet size through solute formation or depletion, it is plausible that chemical reactions could also tune droplets into resonance with the trapping laser, resulting in a thermally locked state. As the driving force for resonance shifts to chemical reactions, the droplets may exhibit growth characteristics distinct from those of nonreacting droplets. Importantly, once thermal locking occurs, the light intensity within the droplets should be significantly amplified, which may, in turn, influence the chemical reaction that initially induced thermal locking, as many key reactions are sensitive to irradiation effects.

With the goal to probe how a chemically reacting droplet interacts with a trapping laser, we designed AOT experiments in which single aqueous droplets were levitated by a dual beam counter propagating optical trap. The growth of the droplet was driven by environmentally important sulfate formation mechanisms, including (a) heterogeneous oxidation of SO_2_ by NO_2_ [18–21] and (b) SO_2_ oxidation by O_2_ with Mn^2+^ as a catalyst [22–24]. In experiments conducted under a range of different conditions, we observed resonance-driven discrete or semi-discrete droplet growth despite continuous sulfate formation. This growth exhibited a distinctive pattern, different from thermal locking induced by physical factors. Furthermore, we demonstrate that resonance with the trapping laser enhances droplet reactivity by accelerating photoinduced reaction pathways, with this effect being most pronounced near the droplet surface.

## Results and Discussion

### Discrete droplet growth

**Fig. 1.**
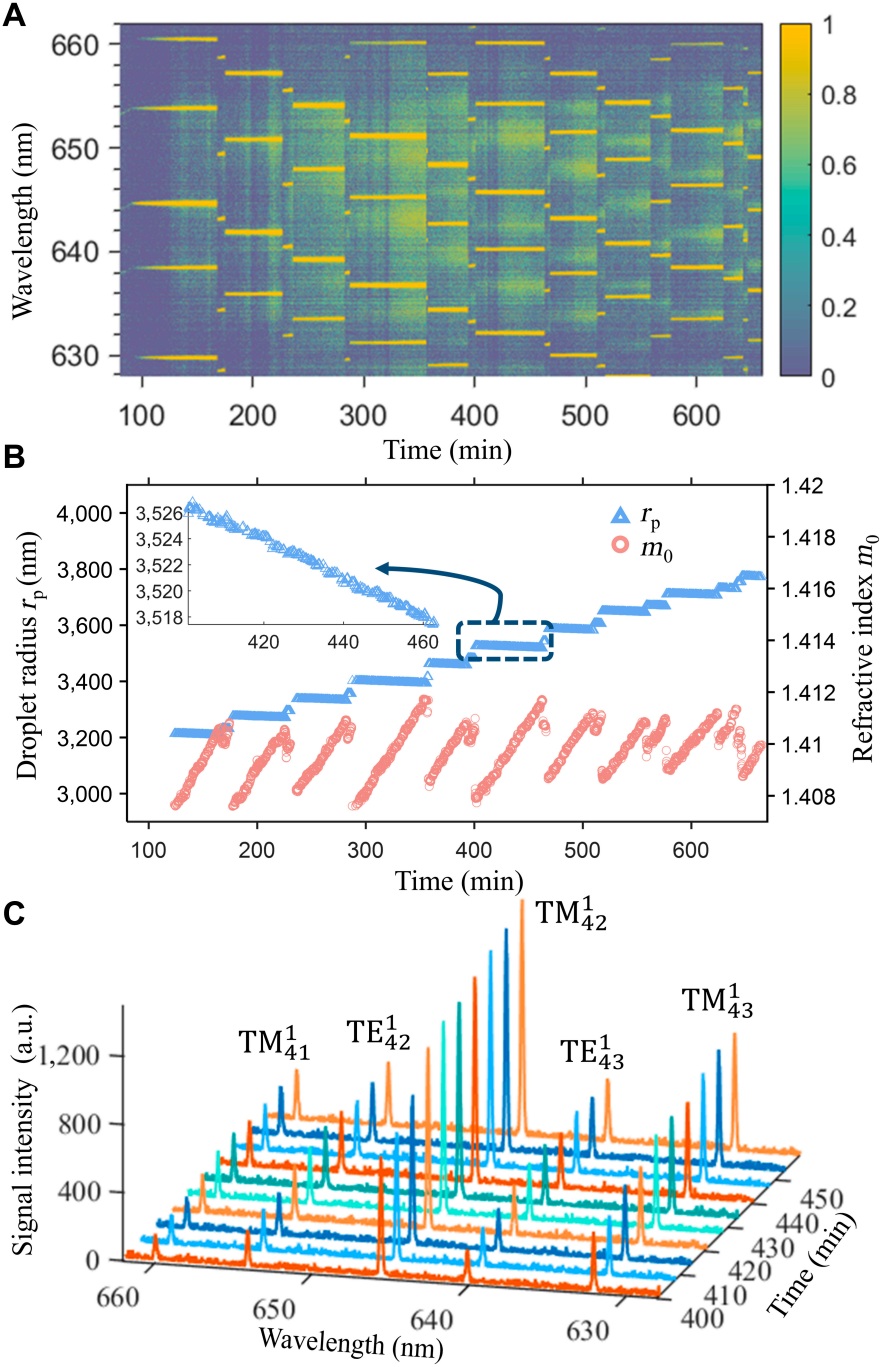
(A) Raman spectra from a representative experiment at RH = 75.7% ±2.0%, [SO_2_] = 0.3 ppm, [NO_2_] = 0.5 ppm, [NH_3_] = 7.6 ppm and an ambient temperature of 24 °C. The *x*-axis shows the time since the start of the measurement, but only the spectra after the RH is completely stabilized are shown to rule out the interference from RH variation. The bright yellow lines correspond to the positions of the whispering gallery modes (WGMs). The signal intensity is normalized to its maximum value in each individual spectrum. (B) The droplet radius *r*_p_ (blue trace) and the real part of the refractive index *m*_0_ (red trace) derived from the WGM positions. The inset figure shows a zoomed-in view of the evolution of the droplet radius within a thermally locked state. (C) The increasing WGM peak intensities within a thermally locked state. The peaks are labeled by the mode order (superscript), mode number (subscript), and polarization (TE or TM).

Figure 1A shows the evolution of the Raman spectra of a levitated, aqueous ammonium sulfate (AS) droplet exposed to SO_2_, NO_2_, and NH_3_ in the wavelength range of 628.0 to 662.0 nm, where the spontaneous Raman peak corresponding to OH stretching is located. The spontaneous Raman scattering is weak but can resonate with the droplet by total internal reflection to further stimulate Raman scattering at wavelengths commensurate with the WGMs. The stimulated Raman scattering (or WGMs) is visible as bright stripes in Fig. 1A and exhibits periods of stable positions followed by sudden transitions to new positions. From fitting the WGM peak positions using the Mie Resonance Fitting (MRFIT) program [25], we obtained the droplet radius *r*_p_ and the real part of the refractive index *m*_0_ at 645 nm. The droplet radius (blue trace in Fig. 1B) shows an overall growing trend, consistent with droplet growth driven by sulfate formation through the SO_2_-NO_2_ heterogenous reaction [18,26]. Akin to the WGM peaks, the droplet size undergoes relatively stable periods followed by abrupt shifts. This pattern contrasts with experimental observations where droplet size changes continuously [8,27], but is similar to the “thermal locking” phenomena observed in previous AOT experiments when the ambient RH or the trapping laser power was varied [14,15]. As the droplet enters a thermally locked state, the droplet temperature increases due to resonant absorption of the trapping laser beam, which enhances water evaporation rates and inhibits droplet growth. When the droplet escapes a locked state, it cools down and quickly grows to a larger size by absorbing ambient water vapor, leading to the apparent discrete growth of the droplet. An amplified view of the droplet size during a thermally locked state (inset plot in Fig. 1B) reveals a decrease in droplet radius by a few nanometers. This decrease seems counterintuitive at first sight and is in stark contrast to the slight droplet size increase during thermally locked states observed previously [14,15]. We shall discuss the underlying mechanism for this gradual decrease in the following sections. (Note that “thermal locking” is conventionally defined as constant droplet size [14]. Here, we use this term in a slightly broader sense to refer to a droplet locked in the same resonant mode with the trapping laser.)

In contrast to the droplet size, the refractive index *m*_0_ (red trace in Fig. 1B) shows an oscillatory pattern as the droplet goes in and out of thermal locking: *m*_0_ increases in each of the thermally locked states but drops to a lower value when state transition occurs. Since the sulfate concentration is positively correlated with *m*_0_, the increase of *m*_0_ during thermally locked states reflects continued sulfate formation through the SO_2_-NO_2_ heterogenous reaction (*m*_0_ of pure water is 1.331). Once the droplet escapes from a thermally locked state, water absorption decreases the sulfate concentration and *m*_0_ drops again.

Zooming in on the WGM peaks in a thermally locked state (Fig. 1C), we observe that the intensities of the WGM peaks increase with time. As a thermally locked state evolves, more trapping laser power is coupled into the droplet, inducing progressively stronger stimulated Raman scattering at WGM-commensurate wavelengths [15,16,28]. The implication of power coupling for chemical reactions shall be discussed in the following sections.

### Retrieving droplet properties during thermally locked states

As discussed above, both the droplet temperature and sulfate concentration vary during thermally locked states; to obtain these 2 quantities, we developed a droplet mass balance model that considers salt formation and transfer of water between the droplet and the gas phase (Section S3). An order-of-magnitude analysis for representative experimental parameters reveals that for thermal locking to occur, the water vapor concentration at the droplet surface $\left(n_{w,s}\right)$ must approximately equal the ambient water vapor concentration ($n_{w,\infty }$, determined by the RH):

$$n_{w,s}\approx n_{w,\infty }$$ (1)

In other words, water evaporation has to proceed at close to equilibrium so that mass transfer of water between the droplet and the gas is slow during a thermally locked state.

**Fig. 2.**
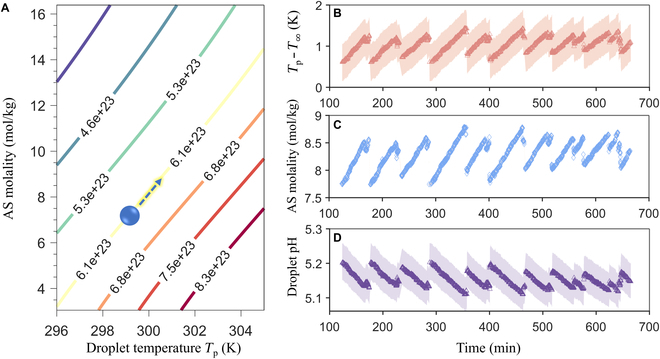
(A) Contour plot of the number density of gas phase water molecules at the droplet surface ($n_{w,s}$ in m^−3^) as a function of AS molality and droplet temperature *T*_p_. The droplet (blue circle) moves along one of the contour lines during a thermally locked state since the ambient water vapor concentration $n_{w,\infty }$ is fixed (Eq. 1). (B) The difference between the droplet temperature *T*_p_ and the ambient temperature $T_{\infty }$. (C) The AS molality of the droplet. (D) The droplet pH. The shaded areas in (B) to (D) correspond to an RH uncertainty of ±2.0% and a $T_{\infty }$ uncertainty of ±0.5 K. The uncertainty of AS molality is only ±0.02 mol/kg and is barely visible in (C).

The water vapor concentration at the droplet surface $\;n_{w,s}$ is a function of both sulfate concentration and droplet temperature. As no detectable nitrate accumulation in the droplet was observed in our experiment (Section S4), we calculated $\;n_{w,s}$ using Extended Aerosol Inorganics Model (E-AIM) IV [29] assuming that the droplet is an aqueous solution of AS. The contour lines of $\;n_{w,s}$ thus obtained are shown in Fig. 2A. Since $n_{w,\infty }$ remains constant during each experiment, Eq. 1 implies that $\;n_{w,s}$ must also remain constant within any thermally locked state. In other words, the AS concentration and the droplet temperature are constrained to evolve along one of the contour lines in Fig. 2A. In addition to $\;n_{w,s}$, the relationship between droplet temperature and sulfate concentration is further constrained by *m*_0_ (Fig. 1B). Building on a previous work [30], we derived a quantitative relationship between *m*_0_, the droplet temperature, and the AS molality (Section S5). With the $\;n_{w,s}$ constraint and the *m*_0_ constraint, we solved for the droplet temperature and salt concentration during thermally locked states, as illustrated in Fig. 2B and C. Both the droplet temperature and AS concentration exhibit oscillatory patterns similar to the refractive index *m*_0_. Based on these results, we further calculated the AS mass in the droplet and the droplet pH with the aid of the E-AIM model. As shown in Fig. S4, the AS mass in the droplet increases in each of the locked states, consistent with continued sulfate formation by the SO_2_-NO_2_ reaction despite thermal locking. Although our model explains most of the AS formation, there are slight AS mass discontinuities between consecutive thermally locked states. This deviation from perfect mass closure might be caused by both measurement uncertainties and the inaccuracies of the parametrizations we used in this study (e.g., the *m*_0_–AS molality relation). Further corroboration of the calculated AS molality can potentially be achieved using the ratio-metric method based on spontaneous Raman signals [31–33]. In contrast to droplet temperature and AS molality, Fig. 2D shows that the droplet pH decreases in each locked state, mainly because of the negative correlation between pH and AS concentration. For a droplet, the salt concentration, temperature, and pH are key variables that affect its reactivity [34,35]. In our example experiment, their variations through a thermally locked state are about 1 mol/kg, 1 K, and 0.1, respectively.

### Droplet trajectory in the *r*_p_–*m*_0_ space

**Fig. 3.**
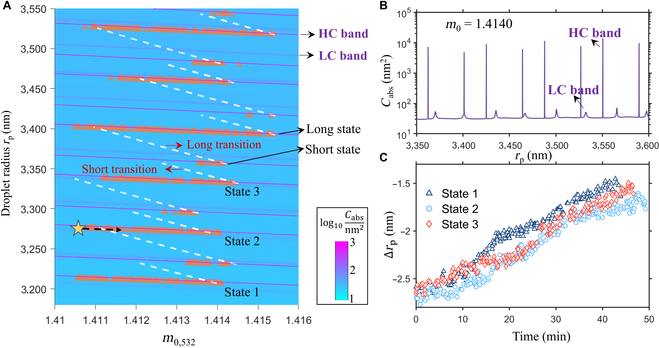
(A) The droplet trajectory in the 2-dimensional droplet size-refractive index space. The heatmap shows the log10 values of the droplet absorption cross-section *C*_abs_ calculated with the GMLT assuming that the imaginary part of the droplet refractive index is 10^−8^. The orange triangles correspond to the experimental data. Two absorption bands and several thermally locked states are labeled to facilitate further discussion. The white dashed lines are simulated droplet trajectories when state transition occurs. Simulation details of the “short transition” and the “long transition” are shown in Fig. S6. (B) The droplet absorption cross-section at *m*_0_ = 1.414. Two *C*_abs_ peaks corresponding to the “HC band” and “LC band” are labeled in (A) and (B). (C) Distance in terms of *r*_p_ between the droplet trajectory and the nearest HC band maximum (Δ*r*_p_) as a function of time after the droplet becomes locked. The distance is shown for 3 thermally locked states labeled as “state 1”, “state 2” and “state 3” in (A).

We next investigate the cause for the gradual reduction of droplet size (inset figure in Fig. 1B) within thermally locked states, which seems counterintuitive since sulfate is continuously being produced in the droplet. Let us consider the droplet trajectory in the size-refractive index (*r*_p_–*m*_0_) space. To do so, we first applied a parametrized salt concentration-dependent dispersion relation [36] to calculate the droplet refractive index at 532 nm (*m*_0,532_) from *m*_0_ at 645 nm (see Fig. 1B). The droplet’s *r*_p_–*m*_0,532_ trajectory during the experiment is then superimposed onto a 2-dimensional heatmap (Fig. 3A) of the droplet absorption cross-section *C*_abs_ at 532 nm (the trapping laser wavelength), which is calculated with the generalized Lorentz Mie theory to account for the Gaussian shape of the trapping laser [37,38]. Figure 3A shows 2 types of absorption bands. The first type is very narrow with high *C*_abs_ maxima (bright purple lines with full width at half maximum on the order of 0.001 nm, referred to as HC band hereafter) and the second type is broader with low *C*_abs_ maxima (darker purple lines with full width at half maximum on the order of 1 nm, referred to as LC band hereafter). The peak heights for the 2 types of bands differ by more than 2 orders of magnitude, as illustrated by a plot of *C*_abs_ at *m*_0_ = 1.4140 (Fig. 3B). Additionally, both the HC and LC bands tilt downwards (toward lower *r*_p_) as *m*_0,532_ increases.

Figure 3A shows that the *r*_p_–*m*_0,532_ data points derived from the WGMs (orange triangles) align closely with the HC bands, suggesting that (a) the absorption of the trapping laser causes thermal locking, and (b) the LC bands are not strong enough to thermally lock the droplet. In the following, we focus on the HC bands and their role in the droplet’s thermally locked state (e.g., the star symbol in Fig. 3A). As sulfate forms through the SO_2_-NO_2_ reaction in the droplet,$\;n_{w,s}$ decreases because of the increasing sulfate concentration (salt effect). The droplet has the tendency to increase in size by absorbing ambient water vapor to re-equilibrate with the environment. However, the HC band acts as a thermal barrier for water absorption by heating the droplet and enhancing water evaporation, disallowing upward movement of the droplet in the *r*_p_ direction. Consequently, sulfate formation only increases *m*_0_ but not *r*_p_, causing the droplet to move along the HC band (the black dashed arrow in state 2 in Fig. 3A).

As the thermally locked droplet moves along just below the HC bands (e.g., states 1 to 3 in Fig. 3A), Fig. 3C shows that the gap Δ*r*_p_ (measured along the *r*_p_ axis) between the droplet trajectory and the HC band maximum gradually decreases by about 1 nm within approximately 50 min. As one of the droplet’s WGMs gets closer to the trapping laser wavelength, more power couples into the droplet, which, in turn, causes its temperature to rise. As shown in Fig. S5, the temperature elevation indeed increases with the decreasing gap. We note that Δ*r*_p_ shown in Fig. 3C is much larger than the width of the HC bands (a few thousandths of nanometers in terms of *r*_p_) but is similar to previously reported values [14,15]. The large difference might be caused by droplet deformation or uncertainties in *r*_p_ and *m*_0_ retrieval from the Raman spectra.

As the droplet escapes a thermally locked state, its temperature drops by conduction and it starts to absorb water so that *m*_0,532_ decreases and its *r*_p_ increases. We applied a droplet growth model to simulate this dynamic process, which considers water vapor mass transfer, condensational heating, and heat conduction between the droplet and gas phase (Section S7). The simulation reveals that the time scale for the transition between the states is on the order of 10 ms, which is much shorter than the time for Raman spectra collection (2 to 3 s) and indicates that high time resolution spectroscopic methods, e.g., broadband light scattering [11], would be required to capture this dynamic transition process. The modeled trajectories during state transition are shown in Fig. 3A by the white dashed lines. The calculated trajectories roughly connect the end points of one state to the start of the next state, although this connection is not perfect due to uncertainties in the retrieved *m*_0_ and *r*_p_ values (see Section S2).

The trajectory in the *r*_p_–*m*_0_ space also shows that the thermally locked states are of different “length” along the *m*_0_ dimension (a “long” state and a “short” state are labeled in Fig. 3A). Short states take place in between long states, and vice versa. The length of a given state might be determined by the *m*_0_ value where droplet heating induced by light absorption is maximal. However, our data do not show a clear correlation between the state lengths and the *C*_abs_ peak values of the corresponding HC bands. Instead, in the example case, the state lengths seem to be related to the distance (in terms of *r*_p_) between neighboring HC bands. The distance between HC bands is positively correlated to the degree of droplet property relaxation when the droplet transitions between the corresponding thermally locked states. Figure S6 shows two of these relaxation processes (corresponding to the “short transition” and “long transition” in Fig. 3A) and demonstrates that if a thermally locked state is preceded by a longer transition process, droplets in this state will likely start at a low *m*_0_ value and cover a longer distance in *m*_0_ before escaping. Although the example experiment shows that the droplet is consecutively captured by the high HC bands, we note that some thermally locked states are short-lived. Therefore, there is a possibility that the droplet may jump over some HC bands without being captured, particularly if the maximum droplet absorption cross-section is changed by, e.g., the degree of laser beam focusing [16].

Previous works exploited physical parameters, e.g., increasing RH or reducing the trapping laser power [12,15,16], to trigger thermal locking of levitated droplets. In these experiments, contrary to Fig. 1B, *r*_p_ stays nearly constant during thermally locked states, suggesting different locking mechanisms from the chemistry-driven case observed here (see Section S8 for an explanation). Our analysis clearly demonstrates that the reduction of *r*_p_ by thermally locked droplets contains information about solute concentration and can be considered as a signature/identifier of droplets going through chemical reactions under laser irradiation.

### Accelerated sulfate formation in thermally locked droplets

**Fig. 4.**
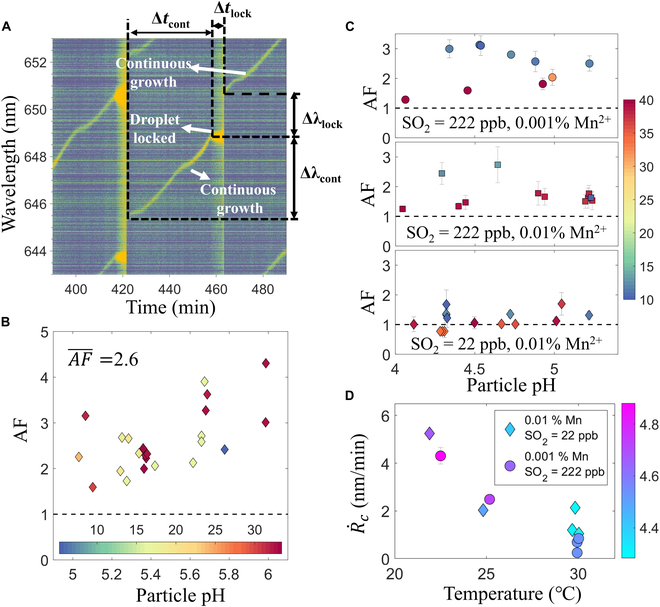
(A) An example Raman spectrum showing the intermittent continuous and discrete change of the WGM observed during an SO_2_-O_2_-Mn^2+^ experiment. $∆\lambda_{\text{lock}}$, $∆t_{\text{lock}}$, $∆\lambda_{\text{cont}}$, and $∆t_{\text{cont}}$ as in Eq. 2 are illustrated in the figure. (B) The acceleration factor AF in the SO_2_-NO_2_ experiments as a function of droplet pH and ionic strength (indicated by color). (C) The measured AF at 3 combinations of SO_2_, Mn^2+^ concentrations as a function of droplet pH. The color bar indicates the ionic strength of the droplets. (D) The corrected droplet growth rate ${\dot{R}}_{c}$ in the SO_2_-Mn^2+^-O_2_ experiments at different temperatures. The color bar indicates the droplet pH.

We next examine the influence of thermal locking on chemical reaction rates. Toward this end, we analyze the Raman spectra that exhibit intermittent continuous and discrete shifts of the WGMs (e.g., Fig. 4A). Such “semi-discrete” WGM shifts indicate that particles consecutively experience continuous size growth and thermal locking. These phenomena are occasionally observed in SO_2_-NO_2_ experiments (none or only a few in experiments that typically last for more than 10 h) but occur in nearly all the SO_2_-O_2_-Mn^2+^ experiments. Although the underlying cause for different particle growth patterns (fully discrete vs. semi-discrete) is still under investigation, the data analysis method presented in preceding sections applies to all thermally locked droplets. More importantly, a comparison of the sulfate formation rate in continuously growing and locked droplets unveils the effect of thermal locking on the investigated reactions.

Since reliable retrieval of the droplet size of unlocked droplets is compromised due to low WGM signal intensities, we employ the following method for the comparison of sulfate formation rates. Figure 4A indicates that within a continuous growth period, the mode wavelength is close to a linear function of time and its rate of change can be approximated by $\Delta \lambda_{\text{cont}}/\Delta t_{\text{cont}}$. This wavelength shifting rate is proportional to sulfate formation rate in the particle [28] (see proof in Section S9). During thermal locking, however, the WGM positions are fixed and their shifts cannot be directly used to indicate sulfate formation. Instead, the wavelength jump $∆\lambda_{\text{lock}}$ (i.e., the difference in wavelength of a WGM before and after thermal locking) is still proportional to changes in droplet size (and hence sulfate content in the droplet) before and right after thermal locking. With these relations, sulfate formation rate during thermal locking can be represented by $∆\lambda_{\text{lock}}/∆t_{\text{lock}}$. Based on the above analysis, we calculate an acceleration factor (AF) with:

$$\begin{array}{c} \begin{array}{c} \begin{array}{c} \mathrm{AF}=\frac{\text{sulfate formation rate in}\;a\;\text{thermally locked state}}{\text{sulfate formation rate in}\;a\;\text{preceding continuous growth period}\;}\\ \;=\frac{∆\lambda_{\text{lock}}/∆t_{\text{lock}}}{∆\lambda_{\text{cont}}/∆t_{\text{cont}}} \end{array} \end{array} \end{array}$$ (2)

The variables in Eq. 2 are illustrated in Fig. 4A and a detailed proof of Eq. 2 is given in Section S9.

Figure 4B shows the AF for the SO_2_-NO_2_ reaction conducted at a variety of conditions. AF quantifies to what extent resonance promotes the droplet reactivity and has an average value of 2.6. AF does not show a clear dependence on droplet pH or ionic strength (indicated by color). Rate acceleration during thermal locking could be caused by either a rise in droplet temperature or photoinduced reactions, which can be significantly enhanced at resonance conditions. To address this, we conducted experiments with fixed concentrations of gas phase reactants but varied temperature by about 6 K in the reaction cell (for reference, thermal locking usually increases the droplet temperature by no more than 2 to 3 K in our experiments, estimated with the method described in Section S5). This set of experiments (Fig. S8) demonstrates that temperature rise does not increase the apparent SO_2_-NO_2_ reaction rate, suggesting that although higher temperature may increase the rate constants between SO_2_ (more specifically, HSO_3_^−^ and SO_3_^2−^) and NO_2_, the decreased effective solubility of SO_2_ and NO_2_ have slightly more pronounced effects to slow down sulfate formation [39]. The weak dependence of droplet growth rate on temperature leaves enhanced photoinduced reactions as the explanation for the accelerated sulfate formation. Such reactions may be initiated by photons activating NO_2_ molecules to an excited state with higher reactivity [40].

We next examine the AF values for the SO_2_-Mn^2+^-O_2_ experiments. Figure 4C shows AF as functions of droplet pH and ionic strength (indicated by the color) at 3 reaction conditions. At both [SO_2_] = 222 parts per billion (ppb), [Mn^2+^] = 0.01% and [SO_2_] = 222 ppb, [Mn^2+^] = 0.001%, AF have values greater than 1 regardless of the Mn^2+^ concentration [note that Mn^2+^ concentration is indicated by its percentage of the (NH_4_)_2_SO_4_ concentration at the onset of an experiment]. Experiments at [Mn^2+^] = 0.01% even yield a slightly lower average AF compared to those with [Mn^2+^] = 0.001% (1.72 vs. 2.38), indicating that lower Mn^2+^ concentration is not a limiting factor for reaction rate acceleration. In contrast, experiments with [SO_2_] = 22 ppb have reduced AFs with values even slightly below 1, suggesting that SO_2_ availability may constrain rate acceleration. To further test this hypothesis, a set of experiments was conducted with increasing SO_2_ concentrations (Fig. S9). These results indicate that AF increases with SO_2_ concentration, confirming that rate acceleration during thermal locking requires abundant SO_2_.

To further investigate whether the reaction rate acceleration in the SO_2_-Mn^2+^-O_2_ system is driven by temperature or light enhancement effects, we measured the droplet growth rate under both a high AF condition (0.001% Mn^2+^, [SO_2_] = 222 ppb) and a low AF condition (0.01% Mn^2+^, [SO_2_] = 22 ppb) in the temperature range of 295 to 303 K. Figure 4D presents the droplet growth rate ${\dot{R}}_{c}$ at different temperatures. This growth rate is corrected for the dilution of Mn^2+^ as the droplets grow and also excludes the influence of thermal locking on droplet temperature (see details in Section S12); hence, it well represents the temperature effect on sulfate formation rates. Figure 4E reveals that ${\dot{R}}_{c}$ and the droplet pH (calculated using the EAIM model IV) both decrease as temperature increases. This indicates that, while the reaction rate constants for the SO_2_-Mn^2+^-O_2_ system may increase with temperature, the reduced pH at elevated droplet temperatures overwhelms to slow down droplet growth—possibly by decreasing the effective Henry’s constant of SO_2_. The negative impact of temperature on droplet growth implies that the heightened light intensity during thermal locking is indeed responsible for the observed reaction rate acceleration. Additional experiments were also conducted in which the laser power is increased from 200 to 500 mW. These experiments confirm that higher light intensity within the droplets promotes sulfate formation (Fig. S11).

Our experiment results provide some clue on the SO_2_ oxidation mechanism in the SO_2_-Mn^2+^-O_2_ system under intense laser illumination. Previous studies [23,41] and our analysis above suggest that sulfate formation proceeds simultaneously via photoinduced and dark reaction pathways, with the photoinduced reaction playing a dominant role at high AF conditions. Building on prior works [41,42], we hypothesize that the photoinduced reaction initiates with photoexcitation of Mn^3+^ to *Mn^3+^. A plausible subsequent step involves a single electron transfer from $\mathrm{HS}O_{3}^{-}$ to *Mn^3+^, resulting in the formation of the $SO_{3}^{-}$ radical:

Mn3++hv→Mn∗3+ (R1)

Mn∗3++HSO3−→Mn2++SO3∙−+H+ (R2)

The $SO_{3}^{∙-}$ radical further reacts with oxygen to form $SO_{5}^{∙-}$, which oxidizes ${\mathrm{Mn}}^{2+}$ to regenerate ${\mathrm{Mn}}^{3+}\;$and produces sulfate [41]. We note that ${\mathrm{Mn}}^{3+}$ may exist in the form of $\mathrm{Mn}{\left(\mathrm{OH}\right)}_{x}^{3-x}$, while $\mathrm{HS}O_{3}^{-}$ could form a complex with Mn^2+^ as $\mathrm{Mn}{\left(\mathrm{HS}O_{3}\right)}^{+}$. At our experimental pH, $SO_{3}^{2-}$ may also participate in the redox reaction. Reaction R2 supports our observation that low SO_2_ concentration limits photoinduced rate enhancement (Fig. 4C and Fig. S11). It also aligns with the strong increase of droplet growth rate with droplet pH (Fig. S12), since higher pH leads to enhanced SO_2_ dissolution and increases $\mathrm{HS}O_{3}^{-}$ concentration. Furthermore, a close inspection of Fig. 4C indicates that higher ionic strength overall reduces AF (compare data points with different colors), likely due to the interference of high concentrations of ${\mathrm{NH}}_{4}^{+}$ and ${\mathrm{SO}}_{4}^{2-}$, which hinder the interaction between ${\mathrm{Mn}}^{3+}$ and $\mathrm{HS}O_{3}^{-}$. Although R1 and R2 qualitatively explain our observations, we acknowledge that a comprehensive understanding of the photoinduced reaction will require experiments across a broader parameter space (including droplet pH, SO_2_, and Mn^2+^ concentrations) and validation through quantum chemical calculations.

### Locale of photoinduced reactions at resonance conditions

**Fig. 5.**
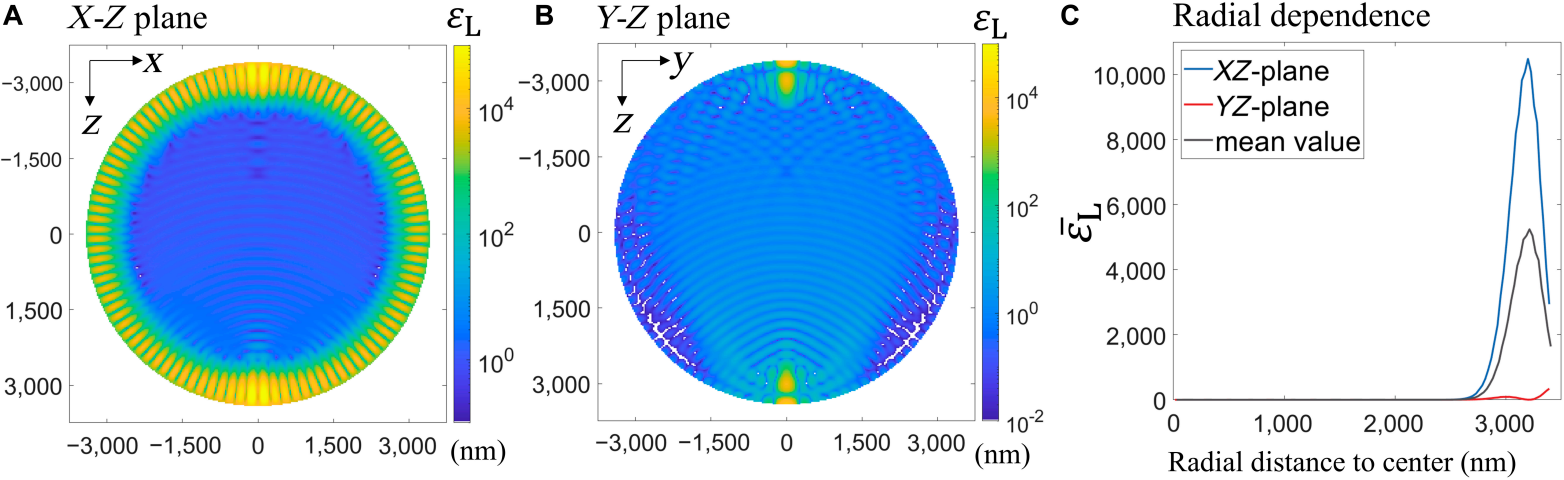
(A and B) The light intensity enhancement factor $\varepsilon_{L}$ in the *X*–*Z* and *Y*–*Z* plane. The incoming light is in the positive *Z* direction and polarized in the *X* direction. The droplet size is 3,407.37 nm in radius and the refractive index is 1.41. The software used for this calculation is PyMieLab_V1.0 [49]. The resonance mode is ${\mathrm{TM}}_{50}^{1}$ and its quality factor is $5.3\times 10^{5}$ based on Mie theory. (C) Angularly averaged $\varepsilon_{L}$ as a function of distance from the droplet center in the *X*–*Z* plane, in the *Y*–*Z* plane, and their mean value.

Our observation of droplet growth is consistent with a droplet growth pattern driven by surface reactions (i.e., reactions at the droplet–gas interface) or reactions occurring in a thin layer beneath the surface, which is manifested by the size independence of the droplet growth rate [8] (Fig. S10). Using an AOT, Cao et al. [22] have shown that without thermal locking, the SO_2_-Mn^2+^-O_2_ reaction occurs on the droplet surface. In our experiment, the size independence of droplet growth rate remains despite thermal locking and high AF values, suggesting that the photoinduced reactions also occur on or in a layer close to the droplet surface. To understand this phenomenon from the perspective of light resonance, we calculated the light intensity enhancement factor ($\varepsilon_{L}$) within a nonabsorbing droplet. $\varepsilon_{L}$ is defined as the ratio of the light intensity within the droplets and the incoming laser. As light intensity is proportional to photon flux, $\varepsilon_{L}$ also represents the photon flux enhancement. Figure 5A and B show an example calculation of $\varepsilon_{L}$ in the *X*–*Z* and the *Y*–*Z* plane, respectively, for an incoming plane wave (at 532 nm) polarized in the *X* direction. The droplet radius and refractive index are 3,407.37 nm and 1.41, respectively, and the resonance mode is ${\mathrm{TM}}_{50}^{1}$. In the *X*–*Z* plane, $\varepsilon_{L}$ is substantially elevated within a few hundred nanometers to the droplet surface, reaching above 10^4^, while in the *Y*–*Z* plane, $\varepsilon_{L}$ is much lower except at regions where it intercepts the *X*–*Z* plane. As shown in Fig. S13, when the ${\mathrm{TE}}_{50}^{1}$ mode resonates, it is in the *Y*–*Z* plane that light intensity is considerably enhanced.

Figure 5C further shows the radial dependence of $\varepsilon_{L}$ (averaged over angles) for the 2 planes as well as their mean value, which is a rough indicator for radial dependence of $\varepsilon_{L}$ for the whole droplet. The radial variation of $\varepsilon_{L}$ suggests that at resonance conditions, photoinduced reactions proceed at considerably faster rates close to the droplet surface and can cause the droplet to exhibit surface reaction characteristics. We note that although elevated $\varepsilon_{L}$ is localized near the droplet surface on a particular plane, it leads to an overall sulfate content increase throughout the droplet rather than only at the $\varepsilon_{L}$ hotspots: on the one hand, the diffusion time scale for the micro-sized, liquid droplets is in the submillisecond range, which is much shorter than the time scale for the micro-sized droplet to increase by 1 nm (which takes at least several seconds in our experiment); on the other hand, the trapped droplet may rotate, leading to a spatial averaging of the light enhancement [43].

Volume integration of light intensity (Fig. S14) reveals that even the droplet-averaged $\varepsilon_{L}$ can reach several hundred under resonance conditions (626 for the case shown in Fig. 5C). At first glance, this high value appears to contradict our experimental observation that increasing laser power by only a factor of 2.5 noticeably enhances sulfate formation at some conditions (Fig. S11C): If the photoinduced sulfate formation rate were directly proportional to light intensity, one would expect higher AF under resonance conditions, where light intensity is amplified by several hundredfold, rather than the observed typical AF of 2 to 3. This apparent discrepancy likely stems from the multistep nature of the sulfate formation mechanism [18,41]: while photons participate in one or a subset of the elementary reactions (e.g., R1 and R2), their impact on the overall reaction rate is constrained by other rate-limiting factors. Indeed, Fig. 5C demonstrates that rate acceleration is conditional, with factors beyond irradiation also playing a critical role in controlling sulfate formation.

## Conclusion

Within droplets and aerosol particles, the rates of a wide range of chemical reactions accelerate by orders of magnitude compared with their rates in bulk solutions [44]. By stably levitating single droplets or aerosol particles up to days under well-controlled conditions, AOTs offer the possibility to examine the rate enhancement of very slow reactions and correlate the reaction rates with precisely measured droplet size. Compared with other single droplet techniques, e.g., electrodynamic balance [45] and acoustic levitation [46], AOT also holds the advantage of characterizing droplets in the submicron range [11,47]. However, using light for droplet levitation can be both a blessing and a curse. On the one hand, elastic and inelastic scattering of the trapping laser enables precise measurements of droplet size, refractive index, and (semi-)quantitative measurement of droplet composition [4]. On the other hand, the intense laser illumination may interfere with chemical reactions or work together with chemical reactions to induce thermal locking, which can obscure droplet growth and challenge data interpretation. Here, we have demonstrated that with a model tailored to our experiment system, it is possible to retrieve the evolution of the droplet properties despite the occurrence of thermal locking. This possibility facilitates wider applications of AOTs to characterize chemically reacting droplets.

The interaction between the trapping laser and reacting droplets exhibits a unique character: while the thermal locking induced by physical means tend to show minute *r*_p_ and *m*_0_ variations within locked states, chemistry-driven thermal locking shows comparatively pronounced droplet property changes. This variation contains information about solute concentration and can be considered as a signature/identifier of droplets going through chemical reactions. To fully understand the behavior of a reacting droplet, we emphasize that the droplet trajectory needs to be analyzed in the 2-dimensional size-refractive index space as opposed to the one-dimensional size space. With an in-depth understanding of the underlying mechanism, it is conceivable to actively utilize thermal locking as a tool to manipulate droplets or aerosol particles. For instance, by tuning the droplet into a thermally locked state, one can use thermal locking to hold the droplet size [12], to obtain strong stimulated (anti-)Stokes signals for composition analysis, or study nonlinear optical effects on a single droplet level (e.g., single droplet lasing [48]).

We have also demonstrated that the substantial increase of light intensity within thermally locked droplets alters the path of a chemical reaction, and such effect has a near-surface localization. For both the SO_2_-NO_2_ and SO_2_-Mn^2+^-O_2_ systems, light illumination at 532 nm may have a negligible influence on sulfate formation at atmospheric conditions. However, once irradiated by a laser with high photon flux, photoinduced reactions emerge and can even dominate when the laser resonates with the droplet, leading to accelerated sulfate formation rates. Since the WGMs are localized near the droplet surface, the droplet growth exhibits patterns similar to those driven by surface reactions. Utilizing the localization of light enhancement, one can potentially compare the stimulated Raman signals of thermally locked droplets with the spontaneous Raman signal of nonlocked droplets (which reflects the chemical composition of the droplet interior) to interrogate the droplet surface properties.

## Methods

Detailed description of the methods and materials can be found in the Supplementary Materials, including the AOT setup, droplet generation and gas supply, data processing procedures, and models for retrieving the properties of thermally locked droplets.

## Data Availability

Research data are available from the corresponding author upon reasonable request.

## Supplementary Materials

Supplementary 1 Figs. S1 to S15 Tables S1 and S2 References [50–53]
