# Supplementary material for: Resonance-Driven Discrete Growth and Chemical Reactivity of Optically Levitated Droplets
Source: Research (Wash D C). 2023 Aug 5;8:0813. doi: 10.34133/research.0813 (PMC12322492; doi:10.34133/research.0813)
Supplement: Supplementary 1 — Figs. S1 to S15 Tables S1 and S2 References [50–53] [file research.0813.f1.docx]

SUPPLEMENTARY MATERIALS

# 1. The experimental setup

## *1.1 The aerosol optical tweezer*

Fig. S1A shows the aerosol optical tweezer (AOT) employed in this study. The trapping laser beam, generated by a continuous wave laser at a wavelength of 532 nm (Opus 532, Laser Quantum), went through an optical isolator (Thorlabs, IO-5-532-HP) to prevent light back-propagation into the laser source. The beam underwent expansion through a telescope consisting of two lenses (50 and 250 mm focal length, respectively). Following expansion, the beam was divided into two cross-polarized beams of equal power using a half-wave plate and a beam splitter. Utilizing a series of reflective mirrors and irises, the two beams were redirected to propagate in opposite directions and were focused by aspherical lenses (ASL10142-532, Thorlabs, numerical aperture = 0.145, working distance = 73.7 mm) to create an optical potential well within the droplet trapping cell. The beam waist was measured by a beam profiler (Thorlabs, BP209-VIS/M) using the knife edge method and was about 6.0 $\mu$m. The laser exiting the Opus 532 had powers ranging from 200 mW to 500 mW during the experiments.


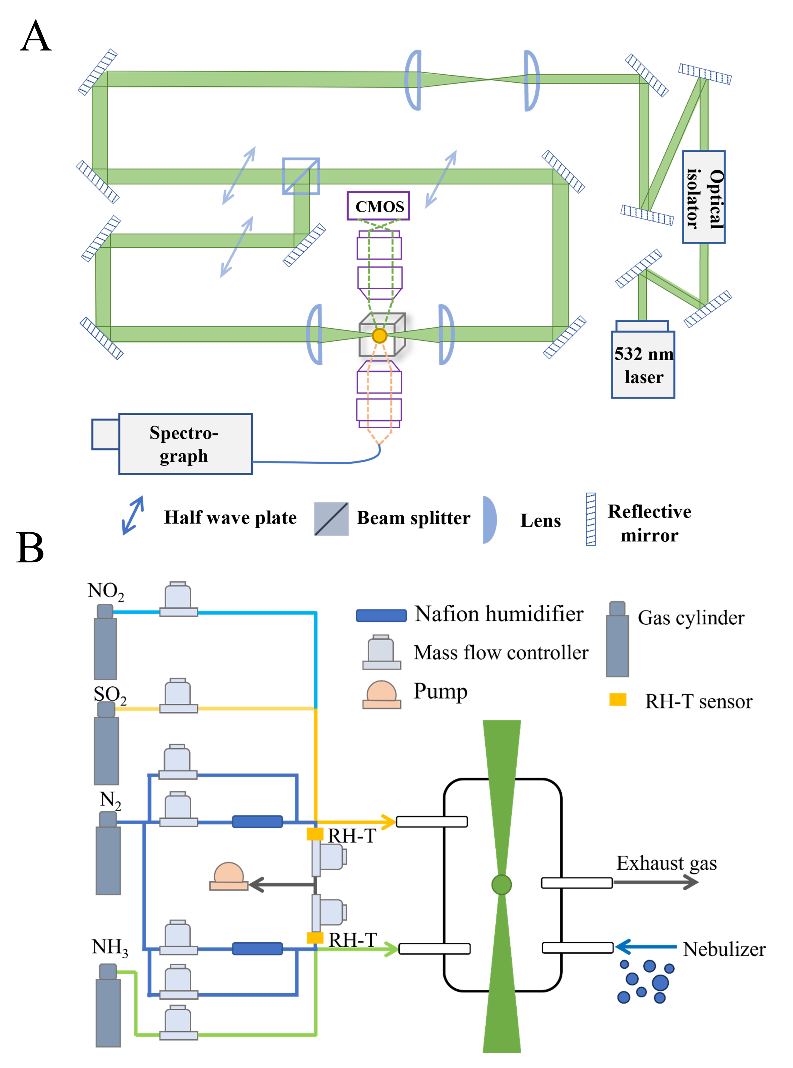


**Fig. S1.** (A) A schematic of the counter propagating optical trap. (B) Schematic of the gas and aerosol supply lines.

Elastically scattered light, collected in the direction perpendicular to the trapping beam, was directed to a CMOS camera (Thorlabs, CS165CU) to monitor the stability of the trapped droplet and ensure that the droplet did not effloresce during low relative humidity experiments. The trapping laser also served as incident light for the inelastic Raman scattering of the levitated droplet. Raman scattering was gathered by a long working distance objective (Mitutoyo, Mitutoyo M Plan Apo 20×Objective), filtered through an OD6 notch filter (for 532 nm), and coupled into an optical fiber by another objective (Olympus, RMS10X). The optical fiber directed the light to a spectrograph (Andor Technology, SR500i) connected with a CCD camera (Newton DU970p-UVB). Before collecting the Raman scattering light, cosmic ray removal and background correction were applied to each Raman spectrum. During signal collection, a 1200 l/mm grating was used to collect light signals in the wavelength range of 627.5-662.7 nm (equivalent to Raman shifts of 2860.7-3707.2 cm^-1^) with an integration time ranging from 2.0 to 3.0 seconds. The spectrograph was periodically calibrated using a Neon (Shanghai Wenyi Optoelectronic Technology Co., Ltd., Ne-1）or a Mercury (Guangzhou Jingyi Optoelectronic Technology Co., Ltd., Hg-1) light source to establish a relationship between the pixel number of the CCD camera and the wavelength.

## *1.2 Droplet generation and gas supply*

In the SO_2_-NO_2_ experiments, the droplets were generated by atomizing a solution prepared with ultrapure water (18.2 MΩ cm) and ammonium sulfate (AS, Sigma Aldrich, ≥99.0%) using a medical nebulizer (Yuyue, Model 405C). In the SO_2_-Mn^2+^-O_2_ experiments, MnSO_4_ (Meryer, AR, 99%) was added to the ammonium sulfate solution (the Mn^2+^ concentration is 0.01% or 0.001% of SO_4_^2-^) prior to atomization. The droplets were transmitted to a custom-made droplet trapping cell through Teflon tubing. The cell, which is cylindrical and has volume extensions to viewing ports, has a volume of approximately 4 cm^3^. It was made of PEEK to minimize chemical reactions on the chamber wall. The droplets in the cell were captured by the laser beam and grew larger through coagulation. Once the captured droplet reached a few micrometers in radius, a clean nitrogen flow was introduced into the cell to sweep away the uncaptured droplets.

The temperature in the trapping cell was controlled by air conditioning in the room and varied by ± 0.5 ℃ during the experiments. The relative humidity (RH) in the cell was controlled at 55-80 % by two nitrogen gas streams. In each gas stream, the RH was controlled by changing the mixing ratio of a dry nitrogen flow and a humidified N_2_ flow (created by passing dry nitrogen through Nafion humidifiers (Perma Pure MH series)). Each of the two gas streams had a flow rate of 100 sccm, but to reduce droplet instability caused by high flow rates, the majority of the flow (84 sccm) was pumped away with a vacuum line before entering the trapping cell. RH and temperature of the two streams were measured with high-precision sensors (Vaisala HM 42). RH and temperature in the trapping cell were calibrated offline against the nitrogen gas streams using another Vaisala HM 42. We estimate that the RH uncertainty was around ± 2.0% during an experiment.

In each experiment, the droplet was exposed to controlled concentrations of SO_2_, NO_2_ and NH_3_. NH_3_ was added to the system to regulate the droplet pH, as low pH impedes the SO_2_-NO_2_ heterogenous reaction(*18*). NH_3_ (3030 ppm in N_2_), SO_2_ (101 ppm mixed in N_2_) and NO_2_ (206 ppm mixed in N_2_) gas cylinders were purchased from Shanghai Weichuang Standard Gas Analytical Technology Co., Ltd. These reactants were introduced into the trapping cell by blending with the humidified N_2_ streams. To avoid reactions (and hence reactant losses) on tube walls by NH_3_, SO_2_ and NO_2_ at high RH, NH_3_ was added to one of the N_2_ gas stream while SO_2_ and NO_2_ were added to the other. Throughout each experiment, the trapping laser power, the RH, and the gas reactant concentrations were fixed.

We observed that it usually took tens of minutes for the humidity in the trapping cell to stabilize to a fixed value with variations less than 1.0%. Therefore, during data analysis we only analyzed the data after the first 100 minutes or so since the trapping of the droplets to avoid interference from a varying RH on droplet size and composition.

# 2. Data processing

The Raman spectra measured by the spectrograph were processed by a custom written MATLAB script. After background reduction, the WGM peaks were identified using the built-in *findpeaks.m* function. These peaks were subsequently fitted with Gaussian distribution functions and the fitted peak positions were used as input to the MRFIT program(*25*). However, during the analysis of some Raman spectra, the extraction of a sufficient number of WGM peaks proved challenging due to either low signal intensity or a noisy signal background. In such instances, the retrieval of r_p_ and m_0_ values from these spectra was unsuccessful, leading to the absence of data points in some of the presented results (see Fig. S15).

The MRFIT program calculates the aerosol radius (r_p_), the refractive index $m_{0}$(real part) at a wavelength $\lambda_{0}$ and the dispersion relation $m_{1}$ using the extracted WGM peaks. In executing the MRFIT program, we assumed that the refractive index $m_{\lambda}$ at a wavelength of $\lambda$ is related to m_0_ by $m_{\lambda}=m_{0}+m_{1}(\frac{1}{\lambda}-\frac{1}{\lambda_{0}})$. Due to uncertainties of the peak positions, the MRFIT program occasionally produced inaccurate droplets properties. For instance, Fig. S2 shows the r_p_ and m_0_ values calculated by the MRFIT program for the representative experiment discussed in the main text. Although most of the retrieved r_p_ and $m_{0}$ follow the expected trend, some data points exhibit large deviations from the overall pattern. We identified these outlier data points using the built-in MATLAB function *isoutlier.m* and excluded them from our presented results (i.e., Fig. 1B in the main text and Fig. S15 in the following). The wavelength residuals (the absolute difference between peak positions calculated by MRFIT and those extracted from the Raman spectra) we obtained from MRFIT is below 10^-2^ nm per peak. For comparison, the uncertainty in peak finding from the measured Raman spectra is about 0.01 nm. Further reduction of wavelength residuals is limited by spectrograph resolution.


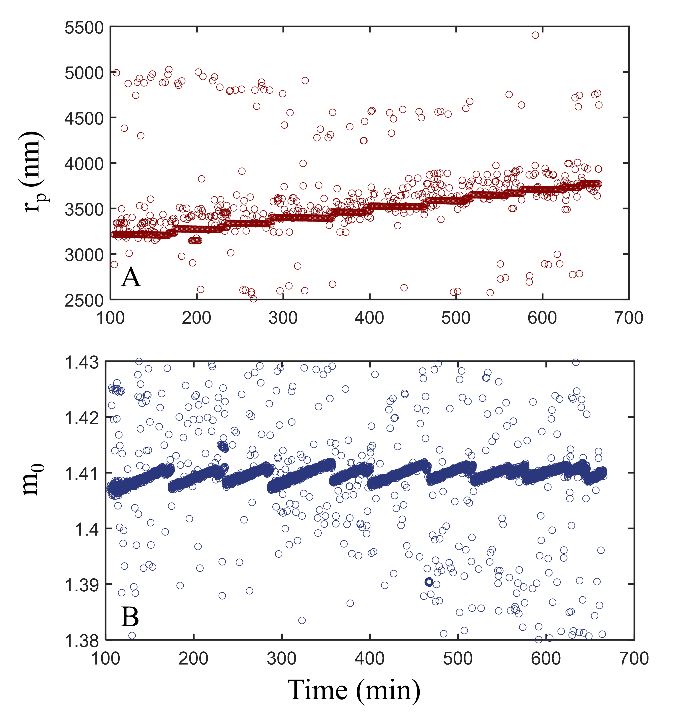


**Fig. S2.** The r_p_ and m_0_ of the droplet as calculated by the MRFIT program without outlier removal in the representative experiment discussed in the main text.

# 3. Droplet mass balance analysis

During a thermally-locked state, the mass change of the trapped droplet can be expressed as

$\frac{dm_{p}}{dt}=4\pi r_{p}D_{w}m_{w}\left( n_{w,\infty}-n_{w,s} \right)+\dot{M}_{AS}$, (S1)

where $m_{p}$ is the droplet mass, $r_{p}$ is the droplet radius, $D_{w}$ is the water vapor diffusivity in nitrogen, $m_{w}$ is the mass of water molecules, $n_{w,\infty}$ is the ambient water vapor concentration, $n_{w,s}$ is water vapor concentration at the droplet surface. The first term on the right-hand side of Equation (S1) is the mass transfer rate of water to the droplet, and the second term $\dot{M}_{AS}$ is the formation rate of AS within the droplet. Substitution of $m_{p}=\frac{4}{3}\pi r_{p}^{3}\rho_{p}$ ($\rho_{p}$ is the droplet density) into Equation (S1) leads to

$\dot{M}_{Salt}={4\pi r}_{p}^{2}\frac{{dr}_{p}}{dt}\rho_{p}-4\pi r_{p}D_{w}m_{w}\left( n_{w,\infty}-n_{w,s} \right)$. (S2)

$r_{p}$ at a given time *t* can be obtained from the MRFIT program (Fig. 1B in the main text) and $\frac{{dr}_{p}}{dt}$ can be calculated by derivatizing $r_{p}$ with respect to *t*. Within a thermally-locked state, both the droplet temperature and AS concentration change over time, making it challenging to solve Equation (S2) because $\dot{M}_{AS}$, $\rho_{p}$, $n_{w,s}$ are dependent on both the temperature and AS concentration of the droplet.

A rearrangement of Equation (S2) leads to the following expression,

$\frac{n_{w,s}}{n_{w,\infty}}= 1+\frac{\dot{M}_{Salt}}{4\pi r_{p}D_{w}m_{w}n_{w,\infty}}-\frac{r_{p}\frac{{dr}_{p}}{dt}\rho_{p}}{D_{w}m_{w}n_{w,\infty}}.$ (S3)

We next evaluate the magnitude of different terms in Equation (S3). Using typical values from our experiments (Table S1), the second and third terms on the right-hand side of Equation (S3) are on the order of 10^-7^ and 10^-8^, respectively. In contrast, a droplet temperature variation of 0.01 K or a molality variation of 0.01 mol/kg causes a change of $\frac{n_{w,s}}{n_{w,\infty}}$ on the order of 10^-4^. This large difference in magnitude means that to deduce the droplet temperature and molality with an accuracy of 0.01K and 0.01mol/kg, the second and third term on the right-hand side of Equation (S3) are negligible. Therefore, for a droplet in a thermally-locked state, Equation (S3) can be simplified to

$n_{w,s}\approx n_{w,\infty}$. (S4)

**Table S1:** Characteristic values the variables in Equation (S3).

| **Quantity** | **Characteristic value** | **Notes** |
| --- | --- | --- |
| $r_{p}$ | $4\times{10}^{-6} m$ | Droplet radii in the experiments range from 2.5-6.5$\times{10}^{-6} m$ |
| $\rho_{p}$ | 1.77$\times{10}^{3}$ kg/m^3^ | This is the density of ammonia sulfate, which is an upper limit of droplet density in the experiment |
| $\frac{{dr}_{p}}{dt}$ | $-2.78\times{10}^{-12} m/s$ | A typical droplet growth rate during a thermally locked state |
| *D*_w_ | $2.53\times{10}^{-5}$ m^2^/s | Water vapor diffusivity at 25 $^{\circ}C$ in nitrogen(*50*) |
| $m_{w}$ | $2.99\times{10}^{-26}$ kg | Mass of a water molecule |
| $n_{w,\infty}$ | $5.39\times{10}^{23}$m^-3^ | Water vapor concentration at 25 $^{\circ}C$, RH = 70% |
| $\dot{M}_{AS}=$  $\frac{\left( \frac{4}{3}\pi r_{p2}^{3}\rho_{p}-\frac{4}{3}\pi r_{p1}^{3}\rho_{p} \right)}{\Delta t}$ | $r_{p1} =4\times{10}^{-6} m$,  $r_{p2} =3.9\times{10}^{-6} m$,  $\Delta t$ = 3600 s | $\dot{M}_{AS}$ is estimated by dividing the difference between the initial droplet mass of two consecutive thermally-locked states by the duration of the first state |

# 4. Droplet composition measurements during SO_2_-NO_2_ reaction

The heterogeneous reaction of NO_2_ with water can lead to the formation of HNO_3_(*51*), which can be further neutralized by NH_3_ and lead to accumulation of NO_3_^-^ ions in the droplet. To verify if nitrate ions exist in the droplets after droplet growth, we measured the Raman spectra in the wavelength range of 541-580 nm (equivalent to Raman shifts of 312.7-1555.6 cm^-1^) during several droplet growth experiments. These measurements were done at high NH_3_ concentrations (~7 ppm) because if NO_3_^-^ were formed in the droplet, the low droplet acidity under high NH_3_ concentrations can promote the NO_3_^-^ to reside in the droplet rather than partition into the gas phase. During these measurements, the Raman spectra showed no obvious NO_3_^-^ peaks (at 1049 cm^-1^) even when droplet grew considerably larger than its original size (Fig. S3A to C).

It is possible that NO_3_^-^ was formed during the above experiments but was not detectable because it had a low concentration. To set a detection limit for NO_3_^-^, we prepared droplets by atomizing a solution containing both (NH_4_)_2_SO_4_ and NH_4_NO_3_, with a molar ratio of (NH4)_2_SO_4_: NH_4_NO_3_ = 10:1. Discernable NO_3_^-^ peaks were observed in the Raman spectra of these droplets at different RH (Fig. S3D to F). This observation implies that in our droplet growth experiment, the highest NO_3_^-^:SO_4_^2-^ ratio in the droplet should be lower than 1:10.

The lack of NO_3_^-^ in the droplet contrasts with chamber experiments performed by Liu et al.(*18*), in which the amount of nitrate formation exceeds sulfate. There are two plausible reasons for such a difference. First, in a chamber environment, the HNO_3_ formed (for instance, through NO_2_ hydrolysis) are confined in the chamber, hence it partitions between the gas and the aerosol particles; in contrast, in our experiments, the droplet is in a constantly flowing nitrogen gas. Nitric acid, if formed at all, may enter the gas phase and is carried away by the flowing nitrogen. Second, we have observed growth of nitrate containing aqueous droplets (NH_4_NO_3_ and NaNO_3_) when only SO_2_ and NH_3_ were introduced into the trapping cell, indicating that the nitrate ions may play a role in oxidizing the SO_2_ to form SO_4_^2-^ under the intense illumination of the 532 nm trapping laser. The exact mechanism for this phenomenon is currently under investigation, but the observation that aqueous NO_3_^-^ ions may promote sulfate formation supports our hypothesis that the droplet mostly consists of (NH_4_)_2_SO_4_.


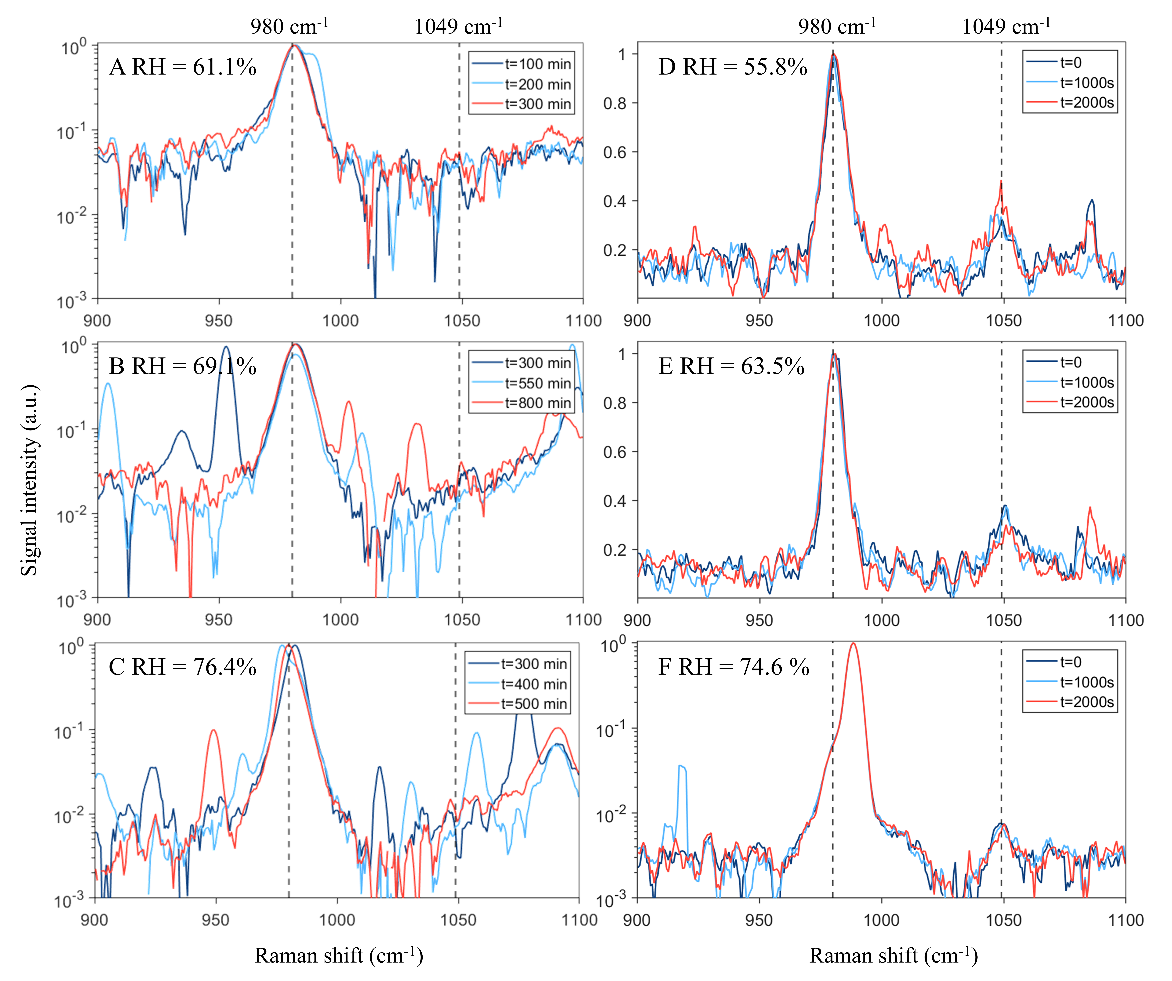


**Fig. S3.** (A-C) The Raman signal of a growing droplet during an SO_2_-NO_2_ sulfate formation experiment at RH = 61.1$\pm$2.0%, 69.1$\pm$2.0% and 76.4$\pm$2.0%. The reaction conditions are [NO_2_]=4.1 ppm, [NH_3_]=6.9 ppm, [SO_2_]=0.5 ppm. (D-F) The Raman signal of mixed (NH_4_)_2_SO_4_-NH_4_NO_3_ droplets at RH = 55.8$\pm$2.0%, 63.5$\pm$2.0% and 74.6$\pm$2.0%. The peak at 980 cm^-1^ corresponds to the SO_4_^2-^ symmetric stretching mode and the peak at 1049 cm^-1^ corresponds to NO_3_^-^ symmetric stretching mode. In panel c, one of the WGM mode overlaps with the spontaneous Raman peak of $\mathrm{SO}_{4}^{2-}$, causing high peak intensity and the peak position to deviate from 980 cm^-1^. In panels d-f, peaks other than the SO_4_^2-^ peak are WGM modes. To better visualize the NO_3_^-^ peak, we used a log y-axis in panels A-C and F.

# 5. Calculation of droplet temperature, salt concentration and AS mass

During a thermally-locked state, we apply two constraints to solve for droplet temperature and salt concentration simultaneously. The first constraint is Equation (S4), which states that the water vapor concentration at the droplet surface is equal to the ambient water vapor concentration. We used E-AIM model IV(*29*) to parameterize the vapor pressure at droplet surface (P_w,s_) as a function of droplet temperature $T_{p}$ and (NH_4_)_2_SO_4_ molality ($C_{\mathrm{AS}})$. Combining the ideal gas law and Equation (S4) leads to the following equation:

$\frac{P_{w,s}\left( T_{p},C_{AS} \right)}{k_{b}T_{p}}=\frac{P_{w,\infty}}{k_{b}T_{\infty}}$, (S5)

where $P_{w,\infty}$ is the ambient water vapor pressure, $T_{\infty}$ is the ambient temperature. The second constraint involves the droplet refractive index, m_0_. m_0_ is negatively correlated with droplet temperature and positively correlated with AS concentration. We assume that the rate of m_0_ change with respect to temperature $\left( \frac{dm_{0}}{\mathrm{dT}} \right)$ is a constant with a value of -$1.25\times{10}^{-4}$/K (*30, 52*), while the $m_{0}$-C_AS_ relation follows the work of Tang et al.(*26*) (corrected using the dispersion relation given by Cotterell et al.(*36*) ) By making an additional assumption that the temperature and salt effect on m_0_ are additive, we have

$\left( T_{p}-T_{\infty} \right)\frac{dm_{0}}{dT}+m_{0}\left( C_{AS}, T_{\infty} \right)=m_{0,m}$, (S6)

where $m_{0,m}$ is the measured refractive index. Using eqs S5 and S6, we can calculate T_p_ and C_AS_ simultaneously.

To further calculate the AS mass within the droplet, the following equation is applied,

$M_{AS}=\frac{4}{3}\pi r_{p}^{3}\frac{r_{AS-W}}{1+r_{AS-W}}\rho_{p}$ (S7)

where $r_{AS-W}$ is mass ratio between AS and water and $\rho_{p}$ is the droplet density. $r_{AS-W}$ is calculated with E-AIM as a function of T_p_ and RH while $\rho_{p}$ is calculated using a correlation between $r_{AS-W}$ and droplet density (also derived with E-AIM). The AS mass within the droplet during the representative experiment is shown in Fig. S4.


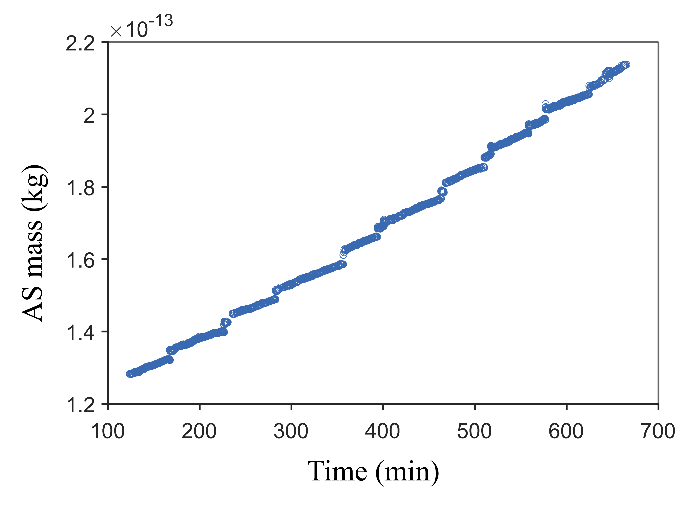


**Fig. S4**. The evolution of the AS mass in the droplet during the experiment discussed in the main text. The discontinuities in AS mass occur when the droplet transitions from one thermally locked state to the next, which might be caused by both measurement uncertainties and the inaccuracies of the parametrizations used in this study (e.g., the m_0_-AS molality relation).

# 6. Droplet temperature elevation as a function of $\boldsymbol{\Delta}\mathbf{r}_{\mathbf{p}}$


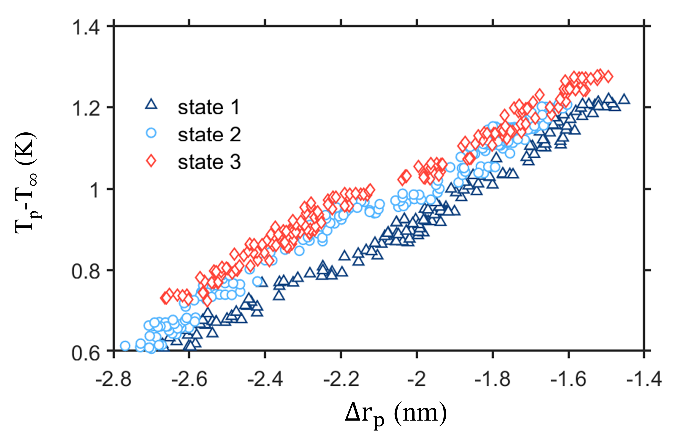


**Fig. S5.** Droplet temperature elevation above the ambient temperature (Tp-T$\text{∞}$) as a function of $\Delta r_{p}$, i.e., the difference between droplet size r_p_ and the nearest HC band maximum (at the same m_0_). Data for three thermally locked states (labelled in Fig. 3A in the main text) are shown.

# 7. Simulating droplet transition between thermally-locked states

To model the transition between the thermally-locked states, we consider heat conduction between the droplet and the gas, water vapor condensation and heat release upon water condensation. The equations that describe these processes are as follows(*39, 53*):

$P_{w,s}=F(C_{AS,}T_{p})$ (S8)

$\frac{dN_{w}}{\mathrm{dt}}=4\pi r_{p}D_{w}(\frac{P_{w,\infty}}{kT_{\infty}}-\frac{P_{w,s}}{kT_{p}})$ (S9)

$\frac{dT_{p}}{\mathrm{dt}}=\frac{\frac{dN_{w}}{\mathrm{dt}}m_{w}L_{w}+ 2\pi r_{p}(T_{\infty}-T_{p})(K_{g}\left( T_{p} \right)+K_{g}\left( T_{\infty} \right))}{M_{AS}c_{p,AS}+M_{w}c_{p,w}}$ (S10)

$F(C_{AS,}T_{p})$ is an interpolation function based on the E-AIM model IV and computes the droplet surface water vapor pressure using the AS molality $C_{\mathrm{AS}}$ and the droplet temperature $T_{p}$.$\frac{dN_{w}}{\mathrm{dt}}$ is the rate of change of the water molecule number in the droplet, $m_{w}$ is the mass of a water molecule, $L_{w}$ is the enthalpy of vaporization of water per unit mass, $K_{g}(T)$ is thermal conductivity of nitrogen at temperature T, $M_{\mathrm{AS}}$ and $M_{w}$ are the total mass of AS and water in the droplet, respectively, $c_{p,AS}$ and $c_{p,w}$ are the heat capacity of AS and water, respectively. To solve the differential eqs S9 and S10, the initial values of T_p_, C_AS_, r_p_ are assumed to have the values at the end of the thermally-locked states (calculated using the method presented in Section S5). The simulation terminates when the droplet size reaches the initial droplet size in the next thermally-locked state.


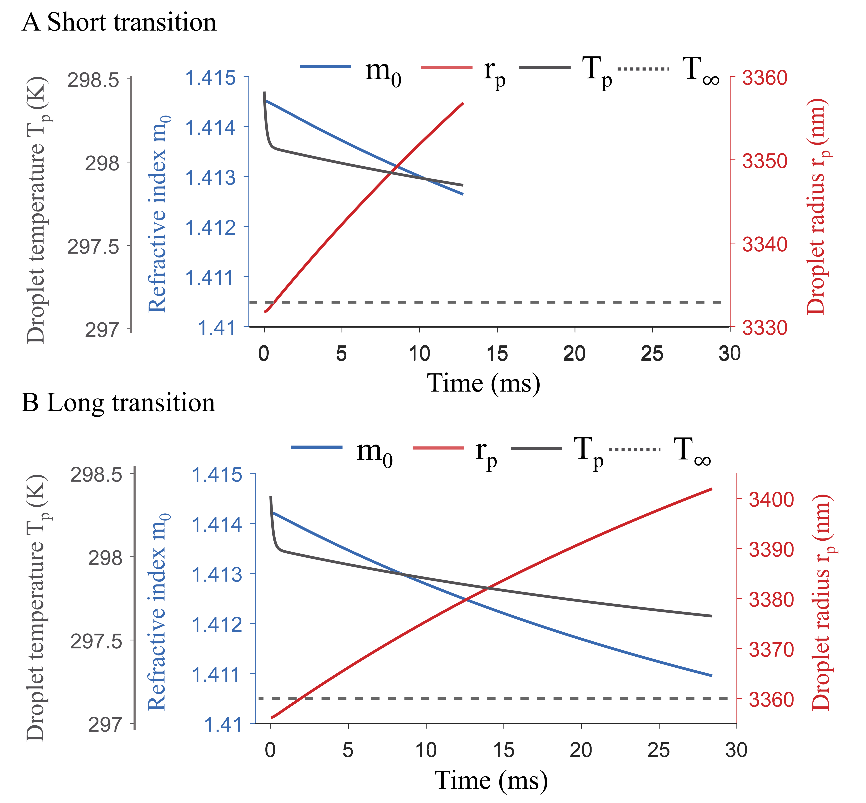


**Fig. S6**. The variation of T_p_, m_0_ and r_p_ during the ‘short transition’ and the ‘long transition’ labelled in Fig. 3A in the main text. The ambient temperature T$\text{∞}$ is shown as a dashed line for reference.

Fig. S6 shows two examples of droplet property evolution during transition between thermally-locked states. Figs. S6A and S6B correspond to the short and long transition labelled in Fig. 3A (main text), respectively. The short transition takes about 13 ms while the long transition takes about 28 ms. As the transition proceeds, the droplet size increases due to water absorption and the refractive index m_0_ decreases due to sulfate dilution. Comparing the m_0_ at the end of the two transition processes, the long transition apparently leads to lower m_0_ values, which implies that the next thermally-locked state will start at a low m_0_ value and is likely to cover a long distance in the m_0_ dimension.

Both heat conduction and heat release by water condensation affect the droplet temperature during transition. In both the short and the long transitions, the droplet temperature evolution consists of a fast-decreasing stage and a slow-decreasing stage. The fast temperature decrease was mainly caused by heat transfer from the droplet to the gas due to conduction (without water condensation, the droplet temperature relaxation time due to heat conduction alone is on the order of a few milliseconds for aqueous droplets with radii of a few micrometers), while the slow decrease reflects the combined effect of heat gain from water condensation and heat loss due to conduction. The droplet temperature does not fully relax to the ambient temperature at the end of both transitions, although it approaches closer to the ambient temperature in the long transition.

# 8. Droplet trajectory analysis as RH increases

Here we provide a qualitative explanation of why the droplet size tends to show minute change during RH-induced thermally locked states. Fig. S7 shows an illustration of the droplet trajectory when the ambient RH increases. The pseudo-color plot is a clip of Fig. 3A in the main text. Let us assume that the droplet is initially located at the red point. As RH increases, if the droplet follows the HC band to the yellow point, its size change is about $\Delta r_{p}\approx11.5$nm and its m_0_ change is about $\Delta m_{0}\approx-0.004$. Such a droplet size increase corresponds to a volume change of 1.04% and an AS concentration decrease of 1.03%. For an AS aqueous droplet with a molality of e.g., 10 mol/kg , the change of molality is approximately 0.1 mol/kg, which corresponds to a droplet refractive index change of only 0.0004(*26*). This value is an order or magnitude smaller than 0.004 and the rest of the m_0_ shift must be explained by the droplet temperature rise. If the value of $\frac{dm_{0}}{dT}$ is -$1.25\times{10}^{-4}$/K(*52*), the droplet temperature needs to increase by approximately $\frac{0.004-0.0004}{1.25\times{10}^{-4}}=28.8 K$. This degree of temperature increase dramatically elevates the water vapor pressure at the droplet surface, which cannot be balanced by the typically minor ambient RH rise observed during RH-induced thermally-locked states (on the order of 1%) (*15, 17*). Therefore, if the droplet moves to the yellow point, the change of droplet properties (droplet size, molality, refractive index) and environmental conditions (RH) are incompatible with one another.


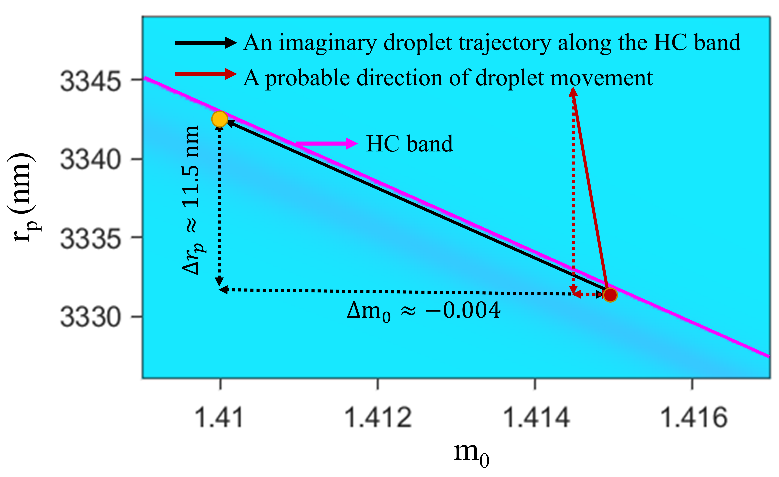


**Fig. S7**. An illustration of droplet trajectories in the r_p_-m_0_ space when the ambient RH increases. Black arrow: an imaginary droplet trajectory that follows the trend of HC band. Red arrow: a probable direction of droplet movement that has larger $\Delta r_{p}:\Delta m_{0}$ ratio than the black arrow.

The red arrow in Fig. S7 shows a more probable direction of droplet movement with a much larger $\Delta r_{p}:\Delta m_{0}$ ratio than the black arrow. Obviously, if the droplet trajectory follows the red arrow, it would be intercepted by HC band with minor changes of r_p_ and m_0_. When this interception occurs, the droplet temperature increases. In contrast to the previous case, the droplet temperature only needs to increase minorly to explain the change of m_0_ (much smaller than the black trajectory) and balance the rising RH. A minor increase of droplet temperature is also consistent with the study conducted by Miles et al.(*15*)

# 9. Relation between sulfate formation and WGM wavelength change

Consider the growth of an unlocked droplet. Under fixed ambient conditions, the sulfate formation rate is proportional to the rate of change of droplet volume, which leads to:

$\dot{S}=C\frac{dV}{dt}=4\pi Cr_{p}^{2}\frac{dr_{p}}{dt}$, (S11)

where C is a constant which is dependent on the ambient temperature, the ambient RH and the type of solute (all fixed during a given experiment). For a given first order WGM mode, the droplet radius is proportional to the mode number, i.e., $r_{p}=\frac{n\lambda}{2\pi N}$.(*28*) This relation together with Equation (S11) leads to

$\dot{S}\propto r_{p}^{2}\frac{d\lambda}{dt}\approx r_{p}^{2}\frac{\Delta\lambda}{\Delta t}$, (S12)

In the above equation, $\Delta\lambda$ is the WGM shift within a time interval $\Delta t$ during which $r_{p}$ only have minor variations. Equation (S12) shows that the WGM wavelength shifting rate is proportional to sulfate formation rates within the droplet. Based on Equation (S12), the ratio $\dot{S}$ at two different time t_2_ and t_1_ is given by:

$\frac{\dot{S}_{2}}{\dot{S}_{1}}\approx\frac{r_{p,2}^{2}\Delta\lambda_{2}/\Delta t_{2}}{r_{p,1}^{2}\Delta\lambda_{1}/\Delta t_{1}}\approx\frac{\Delta\lambda_{2}/\Delta t_{2}}{\Delta\lambda_{1}/\Delta t_{1}}$ (S13)

where second approximation sign holds when $r_{p,2}$ and $r_{p,1}$ are similar in magnitude and thus cancel out.

Following Equation S13, the acceleration factor AF is given by

$AF=\frac{\dot{S} in a thermally locked state}{\dot{S} in a preceding continuous growth period}$ $=\frac{{{(r}_{p} in a thermally locked state)}^{2}\times\Delta\lambda_{lock}/\Delta t_{lock}}{{{(r}_{p} in a a preceding continuous growth period)}^{2}\times\Delta\lambda_{cont}/\Delta t_{cont}}$
$\approx\frac{\Delta\lambda_{lock}/\Delta t_{lock}}{\Delta\lambda_{cont}/\Delta t_{cont}}$ (Equation 2 in the main text)

The final approximation is valid because the square of particle radii $r_{p}^{2}$ in thermally locked state and preceding continuous growth stage are nearly identical (typically differing by only about 1%), allowing their ratio to be treated as unity).

# 10. Effect of temperature on the SO_2_-NO_2_ reaction

**Fig. S8.** The influence of ambient temperature (colorbar) on the droplet growth rate (dr/dt) in the SO_2_-NO_2_ experiment. The SO_2_ and NH_3_ concentrations are 0.5 ppm and 7 ppm, respectively. The NO_2_ concentration are either 2.0 ppm (diamonds) or 0.5 ppm (circles). The experiments were conducted at RH = 80$\pm$2.0%.

# 11. Acceleration factor as a function of SO_2_ concentration


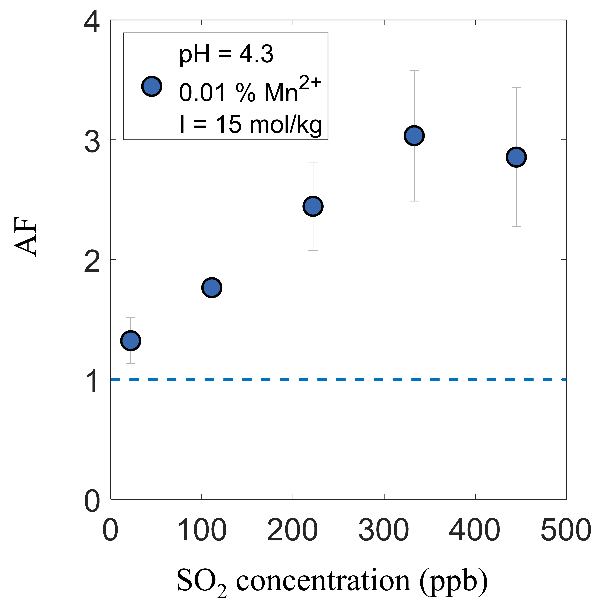


**Fig. S9.** The AF as a function of SO_2_ concentration at droplet pH = 4.3, [Mn^2+^]=0.01% and droplet ionic strength I = 15 mol/kg. The AF increases with SO_2_ concentration until reaching a value about 3.

# 12. Corrected droplet growth rate in SO_2_-Mn^2+^-O_2_ experiments

In the SO_2_-Mn^2+^-O_2_ experiments, the Mn^2+^ concentration within the droplets decreases as the droplet grows. To correct for this effect when analyzing the droplet growth pattern, we multiplied the measured growth rate dr/dt by a volume correction factor V/V_0_ (where V and V_0_ represent the droplet volume during the experiment and at the experiment's onset, respectively). This correction is consistent with first-order reaction kinetics with respect to Mn^2+^ concentration. (Note that in the SO_2_-NO_2_ experiment there is no such dilution of reactants, hence no correction was applied to dr/dt.)

Fig. S10 shows the derived droplet properties at two experimental conditions (see figure caption). To obtain dr/dt, we first fit the droplet radius as a function of time with quadratic and cubic polynomials (panels a and d). Both fittings well describe the evolution of r but yields slightly different dr/dt values (panels b and e). The corrected droplet growth rates, given by $\dot{R}_{c}=V/V_{0}dr/dt$, are also affected by the order of fitting polynomials, but in all cases are close to constant (panels c and f). The constant $\dot{R}_{c}$ indicates that a single $\dot{R}_{c}$ value can be used to represent an experiment.

Previous studies have shown that $\dot{R}_{c}$ is a constant for surface reaction, but is proportional to droplet size if the reaction occur throughout the droplet volume(*22*). Therefore, the size independence of $\dot{R}_{c}$ suggests that sulfate formation predominantly occurs close to the droplet surface(*8*).


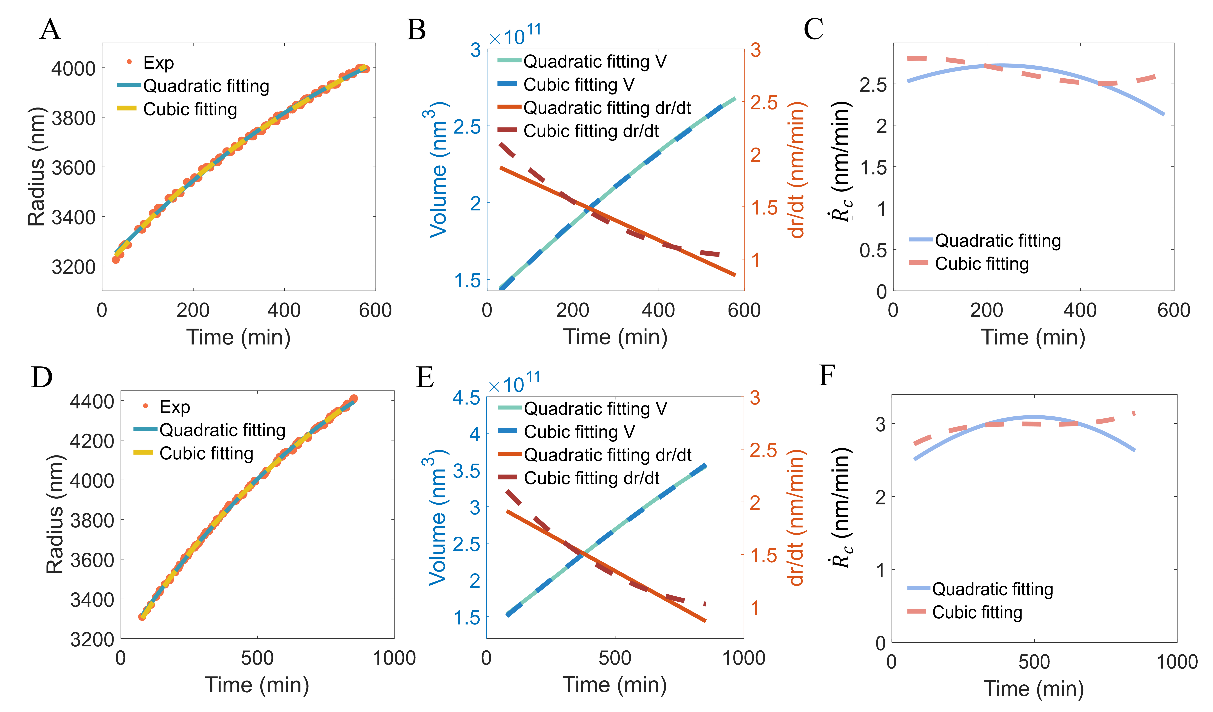


**Fig. S10.** The retrieval of the corrected droplet growth rate ($\dot{R}_{c}=V/V_{0}dr/dt$) from two typical SO_2_-Mn^2+^-O_2_ experiments. The experimental conditions for the upper panels are [SO_2_] = 22 ppb, [NH_3_] = 0.9 ppm, [Mn^2+^]=0.01%, RH = 74.9$\pm$2.0%, T=25.2℃, and conditions for the lower panels are [SO_2_] = 222 ppb, [NH_3_] = 2.3 ppm, [Mn^2+^]=0.001%, RH = 75.5$\pm$2.0%, T=25.2℃. Panels (A) and (D): quadratic and cubic polynomials fittings to the derived droplet size as a function of time. Panels (B) and (D): the droplet volume (left axis) and droplet growth rate (right axis) calculated from the fitting polynomials. Panels (C) and (F): the corrected droplet growth rate $\dot{R}_{c}$ as a function of time.

# 13. Influence of laser power on sulfate formation in the SO_2_-Mn^2+^-O_2_ system


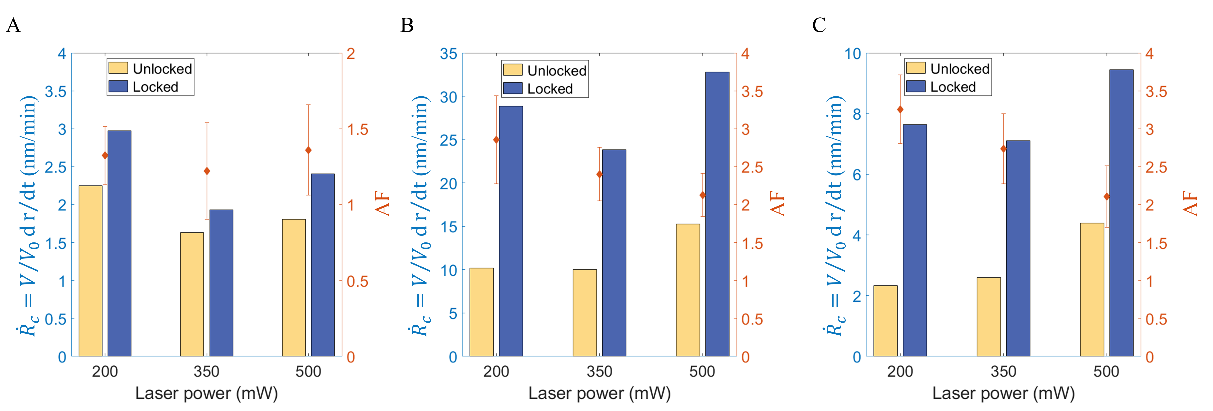


**Fig. S11.** The corrected droplet growth rate $\dot{R}_{c}$ and the sulfate formation enhancement factor at three selected conditions: (A) [SO_2_] = 22 ppb, [Mn^2+^] = 0.01%, pH = 4.3, (B) [SO_2_] = 445 ppb, [Mn^2+^]=0.01%, pH = 4.3, (C) [SO_2_] = 222 ppb, [Mn^2+^] = 0.001%, pH =4.7. To compare the influence of laser power on locked and unlocked droplets, $\dot{R}_{c}$ are separately displayed for these two droplet states with blue and yellow bars. In panel (A), the AF is low, and the $\dot{R}_{c}$ for both locked and unlocked droplets do not exhibit a clear trend with increasing laser power. In panels (B) and (C), the AF is higher. Here, $\dot{R}_{c}$ of unlocked droplets slightly increases with laser power, while $\dot{R}_{c}$ of locked droplets do not have clear trends. This leads to a decreasing AF with laser power (red diamonds). The distinction between panel (A) and panels (B-C) suggests that under low AF conditions, the dark sulfate formation pathway may dominate, resulting in no significant increase in sulfate formation rate when laser power increases. In contrast, for panels (B) and (C), where SO_2_ concentration (445 ppb and 222 ppb) is no longer the main limiting factor of the reaction rate, light-driven pathways can dominate sulfate formation. Consequently, $\dot{R}_{c}$ under non-locked conditions (yellow bars) increases with laser power. In the thermally locked state, however, the photon flux inside the droplet is governed not by incident laser power but by the balance between droplet heating (which is induced by light absorption and drives water evaporation) and salt formation (promoting water condensation). As a result, $\dot{R}_{c}$ during locked-states (blue bars) remains largely insensitive to laser power. Together, these effects explain the observed decline in the acceleration factor (AF) with increasing laser power in (B) and (C).

# 14. Influence of pH on sulfate formation in the SO_2_-Mn^2+^-O_2_ system


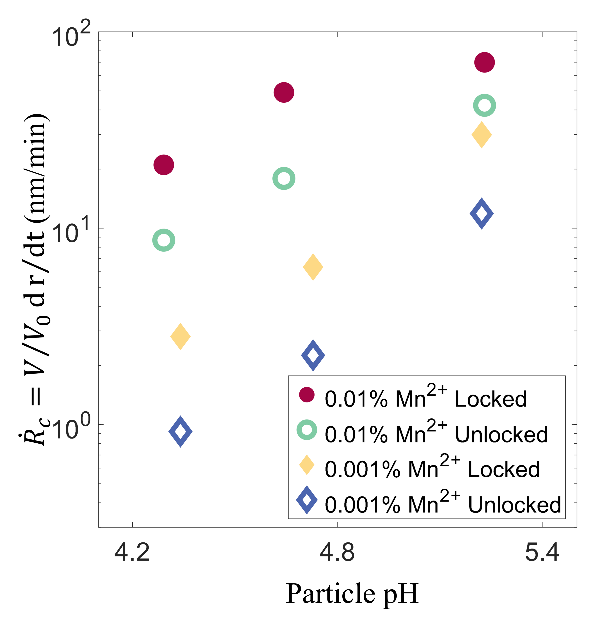


**Fig. S12.** The corrected droplet growth rate $\dot{R}_{c}$ as a function of droplet pH at an SO_2_ concentration of 222 ppb. This figure shows that the sulfate formation rate increases substantially with droplet pH.

# 15. More information on light enhancement

Fig. S13 shows Light enhancement distribution for the $\mathrm{TE}_{50}^{1}$ mode. Combined with Fig. 5 in the main text, it demonstrates how light distribution within the particle changes depending the resonance mode. Fig. S14 shows the average enhancement of light intensity within a layer extending from the droplet surface to the depth indicated on the abscissa. For example, a layer thickness of 100 nm represents the average light intensity enhancement from the surface to a depth of 100 nm within the droplet. When the layer thickness equals the particle radius, the enhancement is averaged over the entire droplet.


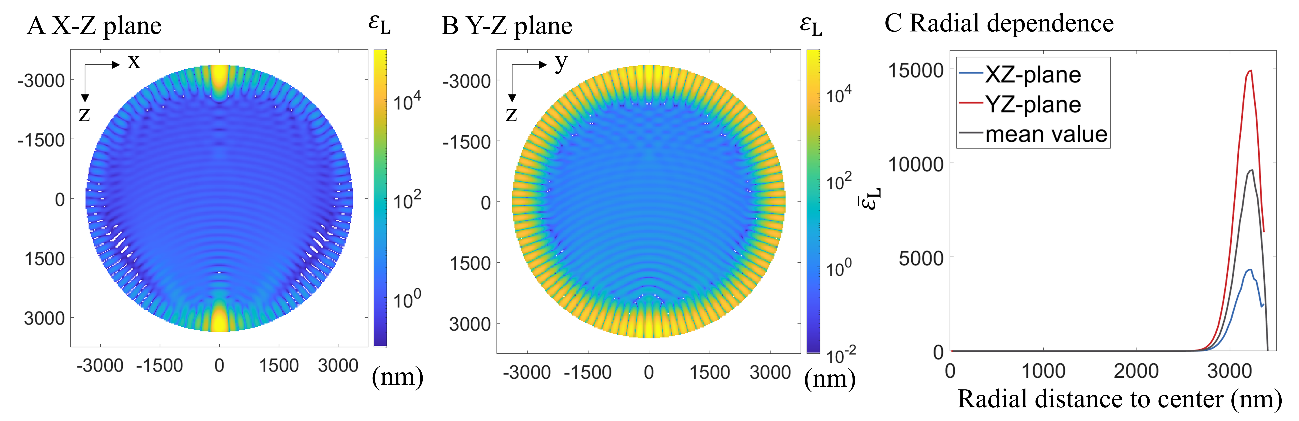


**Fig. S13.** (A) and (B) The light enhancement factor $\varepsilon_{L}$ in X-Z and Y-Z plane. The incoming light is in the positive Z direction and polarized in the Y direction. The droplet size is 3368.93 nm and the refractive index is assumed to be 1.41. The resonance mode is $\mathrm{TE}_{50}^{1}$ and its quality factor is $7.7\times{10}^{5}$ based on Mie theory. (C) Angularly averaged $\varepsilon_{L}$ as a function of distance from the droplet center in the X-Z plane, in the Y-Z plane and their mean value.


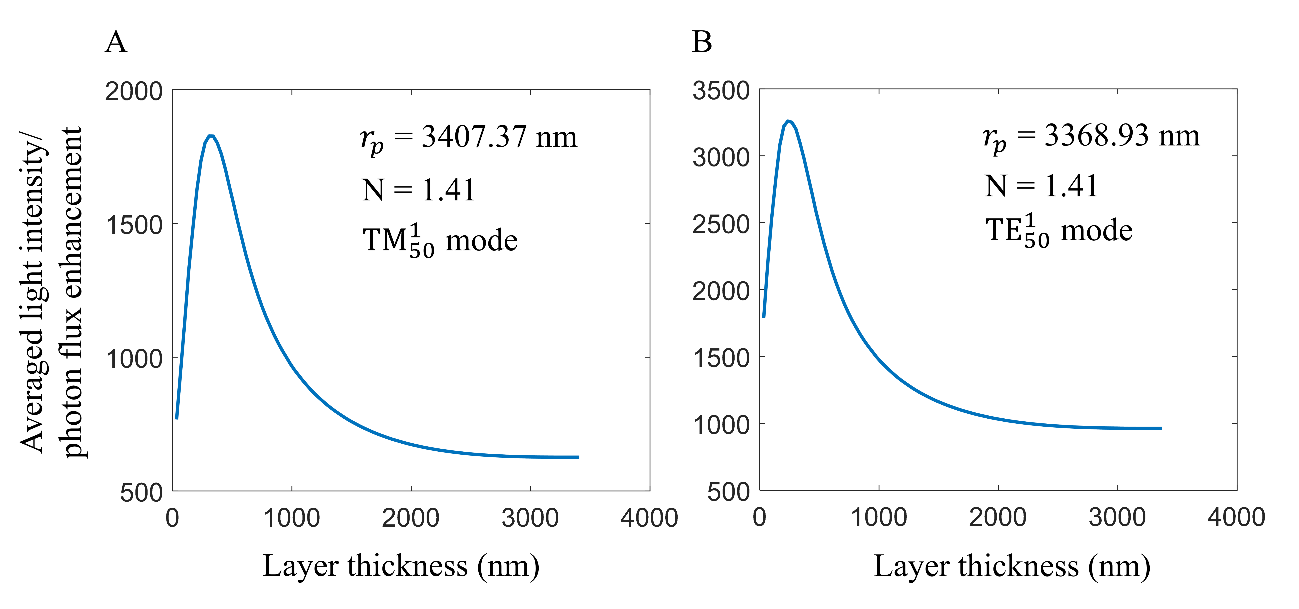


**Fig. S14.** Average light intensity (photon flux) enhancement factor as a function of layer thickness, defined as the distance between the droplet surface and the depth indicated on the abscissa. (A) and (B) correspond to the light intensity distribution shown in Fig. 5 and Fig. S13, respectively.

# 16. Additional experimental results

In addition to the experiment discussed in Fig. 1 in the main text, here we show additional experiments conducted at different reactant gas concentrations and humidity. The experimental conditions are shown in Table S2. The droplet radius, refractive index, temperature, AS molality and droplet pH corresponding in experiments 2-4 are shown in Fig. S15. The overall droplet growth pattern and droplet behavior during thermally-locked states are similar to the experiment discussed in the main text.

**Table S2.** The gas phase reactant concentrations in the experiments. Exp. 1 is discussed in the main text, while results from Exps. 2-4 are shown in Fig. S15.

| **Exp. No.** | **RH (%)** | **SO_2_ (ppm)** | **NO_2_ (ppm)** | **NH_3_ (ppm)** |
| --- | --- | --- | --- | --- |
| 1 | 75.7$\pm$2.0 | 0.3 | 0.5 | 7.6 |
| 2 | 75.7$\pm$2.0 | 4.0 | 0.5 | 7.6 |
| 3 | 75.7$\pm$2.0 | 0.5 | 2.1 | 4.5 |
| 4 | 60.8$\pm$2.0 | 4.0 | 1.0 | 7.6 |


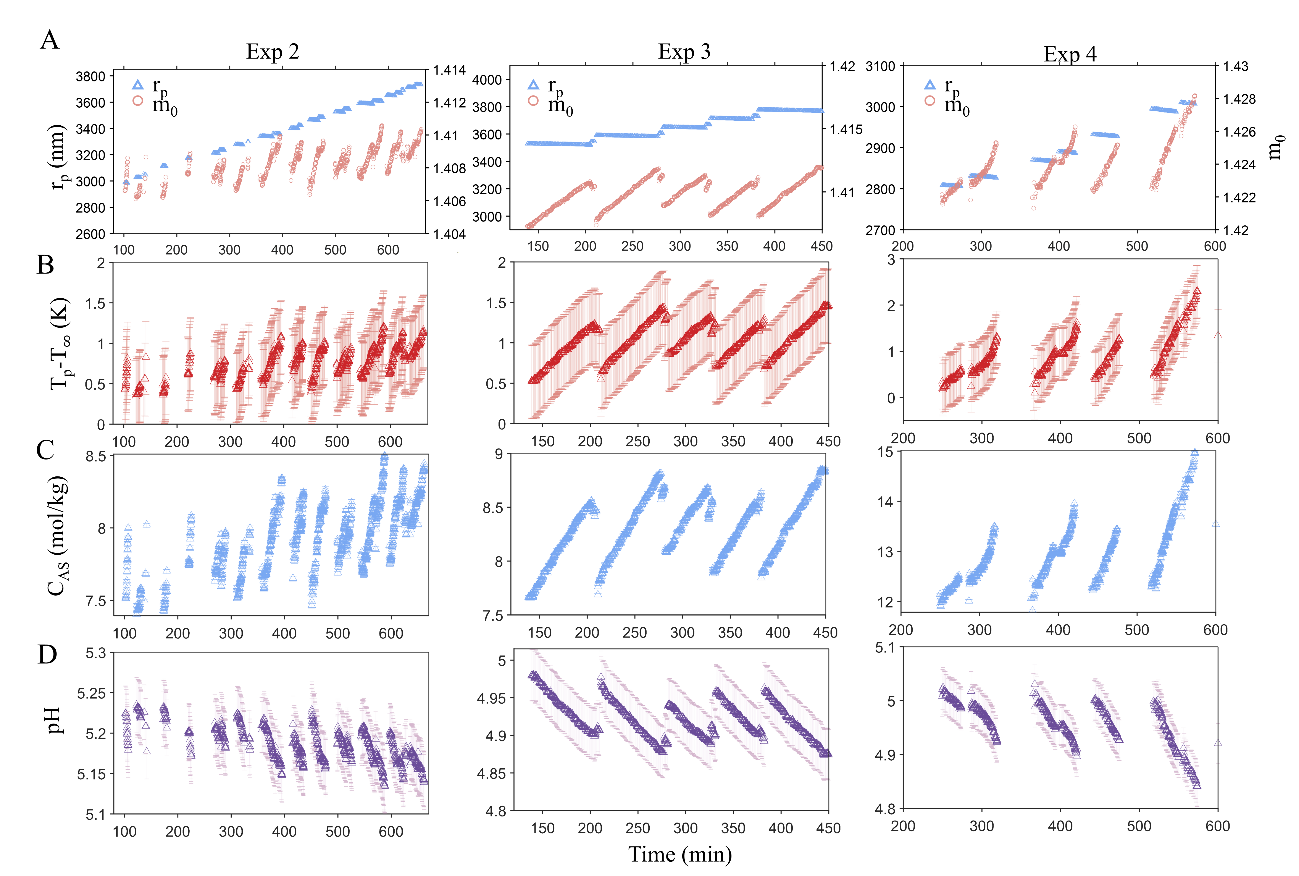


**Fig. S15.** (A) The variation of the droplet radius r_p_ and the real part of the droplet refractive index m_0_, (B) the difference between the droplet temperature T_p_ and the ambient temperature T$\text{∞}$, (C) the AS molality C_AS_, and (D) the droplet pH during experiments 2-4 (conditions listed in Table S2). The error bars correspond to an RH uncertainty of $\pm2.0\%$ and a $T_{\infty}$ uncertainty of $\pm0.5$ K. The uncertainty of AS molality is only about $\pm$0.02 mol/kg and is barely visible in the figure. Data points at certain times are missing because we cannot reliably determine r_p_ and m_0_ from Raman spectra collected at these times (see Sect. S2).
